# COVID-19 mRNA Vaccination and 4-Year All-Cause Mortality Among Adults Aged 18 to 59 Years in France

**DOI:** 10.1001/jamanetworkopen.2025.46822

**Published:** 2025-12-04

**Authors:** Laura Semenzato, Stéphane Le Vu, Jérémie Botton, Marion Bertrand, Marie-Joelle Jabagi, Jérôme Drouin, François Cuenot, Valérie Olié, Rosemary Dray-Spira, Alain Weill, Mahmoud Zureik

**Affiliations:** 1 EPI-PHARE Scientific Interest Group in Epidemiology of Health Products from the French National Agency for the Safety of Medicines and Health Products and the French National Health Insurance, Saint-Denis, France; 2 University Paris-Saclay, Faculté de pharmacie, Orsay, France; 3 University Paris-Saclay, UVSQ, University Paris-Sud, Inserm, Anti-infective evasion and Pharmacoepidemiology Unit/Team, CESP, Montigny le Bretonneux, France

## Abstract

**Importance:**

While several studies have assessed the impact of COVID-19 vaccination on short-term mortality, none have compared long-term mortality by vaccination status, particularly in young individuals who are less likely to experience severe disease following SARS-CoV-2 infection.

**Objective:**

To compare 4-year all-cause mortality in individuals aged 18 to 59 years vaccinated with the mRNA COVID-19 vaccine vs unvaccinated individuals.

**Design, Setting, and Participants:**

This cohort study used data from the French National Health Data System for all individuals in the French population aged 18 to 59 years who were alive on November 1, 2021. Data analysis was conducted from June 2024 to September 2025.

**Exposure:**

Exposure was defined as receiving a first mRNA dose between May 1 and October 31, 2021. Individuals who were unvaccinated by November 1, 2021, were assigned a random index date based on vaccinated individuals’ vaccination dates.

**Main Outcomes and Measures:**

Cox models weighted for sociodemographic characteristics and 41 comorbidities were used to estimate 4-year all-cause mortality. Time to event was censored at all-cause death, COVID-19 vaccination for unexposed individuals, or study termination on March 31, 2025. Complementary analyses were performed, including a comparison of the main causes of death available up to December 31, 2023. Follow-up began 6 months after the index date in both groups to address immortal time bias. Short-term mortality within 6 months after vaccination was assessed in a separate, independent study using adapted self-controlled case series models.

**Results:**

A total of 22 767 546 vaccinated and 5 932 443 unvaccinated individuals were followed up for a median (IQR) of 45 (44-46) months. Vaccinated individuals were older than unvaccinated individuals (mean [SD] age, 38.0 [11.8] years vs 37.1 [11.4] years), more frequently women (11 688 603 [51.3%] vs 2 876 039 [48.5%]) and had more cardiometabolic comorbidities (2 126 250 [9.3%] vs 464 596 [7.8%]). During follow-up, 98 429 (0.4%) and 32 662 (0.6%) all-cause deaths occurred in the vaccinated and unvaccinated groups, respectively. Vaccinated individuals had a 74% lower risk of death from severe COVID-19 (weighted hazard ratio [wHR], 0.26 [95% CI, 0.22-0.30]) and a 25% lower risk of all-cause mortality (wHR, 0.75 [95% CI, 0.75-0.76]), with a similar association observed when excluding severe COVID-19 death. Sensitivity analysis revealed that vaccinated individuals consistently had a lower risk of death, regardless of the cause. Mortality was 29% lower within 6 months following COVID-19 vaccination (relative incidence, 0.71 [95% CI, 0.69-0.73]).

**Conclusions and Relevance:**

In this national cohort study of 28 million individuals, the results found no increased risk of 4-year all-cause mortality in individuals aged 18 to 59 years vaccinated against COVID-19, further supporting the safety of the mRNA vaccines that are widely used worldwide.

## Key Points

**Question:**

Are COVID-19 mRNA vaccines associated with the long-term risk of all-cause mortality?

**Findings:**

In this cohort study including 22.7 million vaccinated individuals and 5.9 million unvaccinated individuals, vaccinated individuals had a 74% lower risk of death from severe COVID-19 and no increased risk of all-cause mortality over a median follow-up of 45 months.

**Meaning:**

These national-level results found no increased risk of 4-year all-cause mortality in individuals aged 18 to 59 years vaccinated against COVID-19, further supporting the safety of the mRNA vaccines that are being widely used worldwide.

## Teaser

This cohort study uses the data from all adults aged 18 to 59 years living in France on November 1, 2021, to evaluate whether there is an association of receipt of an mRNA COVID-19 vaccine with long-term all-cause mortality.

## Introduction

The COVID-19 pandemic, which by early January 2025 has resulted in more than 7 million deaths worldwide, including nearly 170 000 in France, has had a devastating impact on global health, reversing the trend of increasing life expectancy observed over the past decade.^1^ Vaccination has played a central role in mitigating the impact of the COVID-19 pandemic, saving millions of lives globally.^2,3^ Several ecological studies have reported a significant correlation between higher COVID-19 vaccination coverage and lower all-cause mortality rates.^4,5,6,7^ While the protective effect of vaccines on COVID-19 mortality is well-established,^8^ concerns remain about long-term vaccine safety.

Serious adverse events have been reported shortly after vaccination. The main serious adverse events reported following mRNA vaccination were myocarditis, anaphylaxis, and transverse myelitis,^9,10^ while no association was found with the risk of myocardial infarction, pulmonary embolism, or stroke.^11,12^ These events remained rare relative to the number of individuals vaccinated and were predominantly nonfatal.

Several pharmaco-epidemiological studies, primarily using self-controlled case series (SCCS) models, have investigated the short-term risk of all-cause mortality following COVID-19 vaccination.^13,14,15,16,17,18,19,20,21^ They report a significant reduction in non–COVID-19 mortality among vaccinated individuals, which may suggest a protective effect of vaccination against long COVID-19^22^ and/or underreporting of deaths related to undiagnosed SARS-CoV-2 infection. However, unmeasured confounders or healthy vaccinee bias may also contribute to this observed protective effect. This implies that comparisons between vaccinated and unvaccinated groups require specific methodological precautions.

Long-term mortality has not been studied, to our knowledge, particularly among young individuals who are less likely to experience severe disease following SARS-CoV-2 infection. Our objective was to assess the risk of all-cause mortality at 4 years in individuals aged 18 to 59 years who received at least 1 dose of an mRNA COVID-19 vaccine compared with those unvaccinated, using nationwide data.

## Methods

### Data Sources

This cohort study was conducted in compliance with the French regulations on access and processing of personal data from the National Health Data System (SNDS). Neither informed consent nor approval from an ethics committee are required to use these data in a study. The reporting of this study followed the Strengthening the Reporting of Observational Studies in Epidemiology (STROBE) reporting guideline for cohort studies.

French national hospital discharge database (PMSI), coupled with the French national COVID-19 vaccination database (VAC-SI; vaccine products and injection dates) and the SNDS, covers the entire French population (68 million residents). Each person is anonymously identified by a unique, lifelong number. All-cause deaths were identified from civil registry records, available up to March 31, 2025. The SNDS is a set of strictly anonymous databases comprising all mandatory national health insurance reimbursement data, particularly data derived from the processing of health care claims (electronic or paper claims) and data from health care facilities (PMSI). Since 2006, the SNDS has recorded all health care reimbursement data for outpatient care, including drugs, imaging, and laboratory tests, as well as fully reimbursed health expenditures for patients with long-term diseases (LTD), such as cancer and diabetes, while the PMSI has recorded reimbursement data for inpatient care, including diagnoses and procedures performed.

Information on specific causes of death, obtained from the CépiDc registry, was available only up to December 31, 2023, through indirect matching to the SNDS, using date of death, birth month and year, sex, and place of residence, and coded according to the *International Statistical Classification of Diseases and Related Health Problems, Tenth Revision* (*ICD-10*). Their analysis was based on the subset of all deaths occurring before the end of 2023, grouped into categories, as in previous studies.^23^

### Design, Study Population, and Outcomes

Our cohort included alive individuals aged 18 to 59 years, residing in France as of November 1, 2021, and having received any health care reimbursement in 2020 (eg, a medical consultation, dental procedure, medication reimbursement, laboratory tests, hospitalization, and so on). This last selection criterion enabled us to include individuals with similar interactions with the health care system, irrespective of their vaccination status. Exposure to COVID-19 vaccination was defined as the administration of a first dose of an mRNA vaccine between May 1 and October 31, 2021 (inclusion period), which was the mass vaccination period for adults in France, who primarily received mRNA vaccines. Multiple vaccinations in exposed individuals were not considered. The unvaccinated group was defined as individuals who remained unvaccinated as of November 1, 2021. Individuals vaccinated before May 1, 2021 (12.0%), or who received a first dose of another (ie, non-mRNA–based) COVID-19 vaccine during the inclusion period (1.4%) were excluded. Information on vaccination timeline and eligibility criteria in France is available in eMethods 1 in Supplement 1. While the index date for vaccinated individuals corresponds to the date of their first vaccine dose, unvaccinated individuals lack a comparable index date. An index date was randomly assigned to each unvaccinated individual by replicating the distribution of first vaccination dates observed among vaccinated individuals. For all individuals, vaccinated or not, follow-up time zero began 6 months after the index date. This approach was intended to avoid immortal time bias, as unvaccinated individuals were, by definition, alive on November 1, 2021, and to ensure a comparable follow-up duration based on exposure, since inclusion period for the vaccinated group was 6 months. The follow-up duration reported in the results included the 6-month grace period between the index date and time zero. Time to event was censored at all-cause death, administration of a COVID-19 vaccine for unexposed individuals, or study termination on March 31, 2025, whichever occurred first. A summary table of the methodological aspects of our emulated target trial is available in eMethods 2 in Supplement 1.

We also assessed the difference in mortality within 6 months following vaccination in an independent, dedicated study, using the most widely used method for evaluating short-term vaccine safety,^24,25,26^ namely an adapted SCCS model.^27^ The detailed methods are provided in eMethods 3 in Supplement 1.

### Sociodemographic Characteristics, Chronic Diseases, and Comorbidities at Baseline

Sociodemographic variables included age, sex, and region of residence. Age was divided into subgroups of 18 to 29, 30 to 39, 40 to 49, and 50 to 59 years. Socioeconomic variables comprised the area-based social deprivation index^28^ and coverage by the complementary state health insurance (CSS).

We defined comorbidities using the *Cartographie des Pathologies et des Dépenses*,^29^ a set of algorithms based on the reasons for hospitalization, LTD diagnoses, and/or reimbursement of specific treatments in the previous 4 years. This allowed the identification of patients presenting with 41 comorbidities in 2020 (cardiometabolic, respiratory, cancer, inflammatory, neurodegenerative diseases, mental and behavioral disorders, and others). The additional comorbidities of obesity, smoking, and alcohol disorders were also included. History of severe SARS-CoV-2 infection was identified from hospitalization for COVID-19.

### Statistical Analysis

We described the characteristics of vaccinated and unvaccinated individuals. We performed Cox regression models to estimate the incidence of all-cause death during follow-up in individuals vaccinated against COVID-19 with a COVID-19 mRNA vaccine compared with unvaccinated individuals. These models were weighted after modeling the probability of vaccination to standardize incidence using unvaccinated individuals as the reference group (average treatment effect in the untreated). This standardization was based on the month of the index date, sociodemographic characteristics, and the comorbidities presented in Table 1. The individual probability of being vaccinated was estimated using a binomial multivariable logistic regression model^30^ and used to calculate the weights. The weight for unvaccinated patients was set to 1, while for vaccinated patients, it was calculated as the ratio between the probability of being unvaccinated and that of being vaccinated.^31^ We ensured the balance of covariates after weighting by verifying that the absolute value of the standardized mean differences for each variable was less than 0.1 (eFigure 1 in Supplement 1). We provide hazard ratios (HRs) with 95% CIs, derived from the 2.5th and 97.5th percentiles of the estimates obtained through 100 bootstrap resamplings,^32^ generated by random sampling with replacement, so as to obtain the same population size as at inclusion.

**Table 1.** Characteristics at Baseline of Individuals Included in the 4-Year Mortality Study

Characteristic	Participants, No. (%)	
	Vaccinated (n = 22 767 546)	Unvaccinated (n = 5 932 443)
Age, mean (SD), y	38.0 (11.8)	37.1 (11.4)
Age, y		
18-29	6 553 907 (28.8)	1 777 782 (30.0)
30-39	5 535 008 (24.3)	1 737 069 (29.3)
40-49	5 889 066 (25.9)	1 322 270 (22.3)
50-59	4 789 565 (21.0)	1 095 322 (18.5)
Sex		
Male	11 078 943 (48.7)	3 056 404 (51.5)
Female	11 688 603 (51.3)	2 876 039 (48.5)
Regions		
Ile de France	4 555 233 (20.0)	1 065 064 (18.0)
Grand est	1 750 519 (7.7)	435 273 (7.3)
Hauts-de-France	2 105 219 (9.2)	417 622 (7.0)
Auvergne-Rhône-Alpes	2 826 027 (12.4)	722 126 (12.2)
Bourgogne-Franche-Comté	900 018 (4.0)	226 769 (3.8)
Centre-Val-de-Loire	869 428 (3.8)	182 160 (3.1)
Provence-Alpes-Côte d’Azur	1 534 193 (6.7)	669 225 (11.3)
Occitanie	1 937 608 (8.5)	613 934 (10.3)
Nouvelle-Aquitaine	2 036 499 (8.9)	469 027 (7.9)
Normandie	1 152 544 (5.1)	198 833 (3.4)
Pays de la Loire	1 404 548 (6.2)	246 285 (4.2)
Bretagne	1 197 524 (5.3)	210 753 (3.6)
Corse	69 787 (0.3)	33 293 (0.6)
Overseas territories		
Any	428 399 (1.9)	442 079 (7.5)
Guadeloupe	52 665 (0.2)	116 681 (2.0)
Martinique	47 035 (0.2)	104 601 (1.8)
Guyane	25 596 (0.1)	50 705 (0.9)
La Réunion	275 013 (1.2)	157 701 (2.7)
Mayotte	28 090 (0.1)	12 391 (0.2)
Covered by complementary state health insurance	2 087 128 (9.2)	1 240 563 (20.9)
Social deprivation index quintile		
1 (Least deprivation)	4 873 101 (21.4)	929 424 (15.7)
2	4 652 237 (20.4)	1 061 764 (17.9)
3	4 350 793 (19.1)	1 124 831 (19.0)
4	4 158 274 (18.3)	1 089 761 (18.4)
5 (Most deprivation)	4 349 295 (19.1)	1 600 040 (27.0)
Unknown	383 846 (1.7)	126 623 (2.1)
Social security scheme		
General social security scheme	20 461 062 (89.9)	5 414 984 (91.3)
MSA	803 905 (3.5)	207 521 (3.5)
SLM	1 084 198 (4.8)	169 957 (2.9)
Other	418 381 (1.8)	139 981 (2.4)
No. of injections received by time 0		
1	1 846 691 (8.1)	0
2	17 478 998 (76.8)	0
3	3 440 637 (15.1)	0
4	1206 (<0.1)	0
5	12 (<0.1)	0
6	2 (<0.1)	0
Lifestyle habit		
Alcohol addiction	308 333 (1.4)	89 728 (1.5)
Tobacco use	1 142 341 (5.0)	269 832 (4.5)
Comorbidities		
Cardiometabolic		
Any	2 126 250 (9.3)	464 596 (7.8)
Obesity^a^	194 862 (0.9)	39 496 (0.7)
Diabetes	447 044 (2.0)	118 565 (2.0)
Lipid-lowering treatments	540 983 (2.4)	103 374 (1.7)
Hereditary metabolic diseases or amyloidosis	26 600 (0.1)	5881 (0.1)
Hypertension	1 342 076 (5.9)	282 284 (4.8)
Coronary diseases		
Any	128 362 (0.6)	36 875 (0.6)
Obliterating arterial disease of the lower limb	35 701 (0.2)	11 060 (0.2)
Cardiac rhythm or conduction disturbances	84 153 (0.4)	22 428 (0.4)
Heart failure	23 710 (0.1)	8365 (0.1)
Valvular diseases	21 308 (0.1)	6331 (0.1)
Stroke	81 243 (0.4)	24 402 (0.4)
Respiratory diseases		
Chronic respiratory diseases (excluding cystic fibrosis)	752 939 (3.3)	183 137 (3.1)
Cystic fibrosis	1795 (<0.1)	491 (<0.1)
Pulmonary embolism	5890 (<0.1)	1680 (<0.1)
Cancer		
Any	330 482 (1.5)	80 418 (1.4)
Female breast cancer (active)	33 304 (0.2)	8327 (0.3)
Female breast cancer (under surveillance)	55 378 (0.2)	12 021 (0.4)
Colorectal cancer (active)	9504 (<0.1)	2471 (<0.1)
Colorectal cancer (under surveillance)	13 128 (0.1)	3034 (0.1)
Lung cancer (active)	3559 (<0.1)	1213 (<0.1)
Lung cancer (under surveillance)	2857 (<0.1)	849 (<0.1)
Prostate cancer (active)	4463 (<0.1)	1147 (<0.1)
Prostate cancer (under surveillance)	3039 (<0.1)	773 (<0.1)
Other cancers (active)	87 027 (0.4)	22 856 (0.4)
Other cancers (under surveillance)	125 800 (0.6)	29 871 (0.5)
Inflammatory and skin diseases		
Chronic inflammatory bowel diseases	109 291 (0.5)	26 128 (0.4)
Rheumatoid arthritis and related diseases	44 886 (0.2)	12 168 (0.2)
Ankylosing spondylitis and related diseases	75 250 (0.3)	19 059 (0.3)
Psychological and neurodegenerative diseases		
Neurodegenerative diseases	213 484 (0.9)	63 085 (1.1)
Psychiatric disorders starting in childhood	39 220 (0.2)	10 357 (0.2)
Down syndrome	5898 (<0.1)	2193 (<0.1)
Epilepsy	97 646 (0.4)	27 523 (0.5)
Multiple sclerosis	39 577 (0.2)	12 860 (0.2)
Paraplegia	21 704 (0.1)	7967 (0.1)
Myopathy or myasthenia gravis	11 689 (0.1)	3716 (0.1)
Parkinson disease	8031 (<0.1)	1776 (<0.1)
Dementias (including Alzheimer disease)	3469 (<0.1)	1256 (<0.1)
Mental impairment	39 922 (0.2)	9365 (0.2)
Psycholeptic drugs (with or without a disease)	1 793 421 (7.9)	395 473 (6.7)
Antidepressants	1 181 393 (5.2)	226 458 (3.8)
Antipsychotics	357 442 (1.6)	88 714 (1.5)
Anxiolytics	994 166 (4.4)	237 071 (4.0)
Hypnotics	310 519 (1.4)	72 001 (1.2)
Other pathologies		
Hemophilia or severe hemostasis disorders	15 821 (0.1)	4461 (0.1)
HIV infection	60 353 (0.3)	15 167 (0.3)
Liver diseases	91 013 (0.4)	27 742 (0.5)
Chronic hepatitis C	4526 (<0.1)	1716 (<0.1)
Pancreas diseases	42 804 (0.2)	12 373 (0.2)
Chronic dialysis	1875 (<0.1)	1103 (<0.1)
Kidney transplant	5393 (<0.1)	2278 (<0.1)
History of hospitalization for COVID-19	82 269 (0.4)	38 713 (0.7)

Abbreviations: MSA, Agricultural Social Security Fund; SLM, Local Mutual Insurance Section.

^a^ Obesity was defined through *International Statistical Classification of Diseases and Related Health Problems, Tenth Revision* coding for obesity during hospitalization or via a bariatric surgery procedure.

We stratified the results according to age, sex, region, deprivation index, CSS coverage, history of severe COVID-19, history of any disease, type of first dose of mRNA vaccine received, inclusion period (before and after implementation of the vaccine pass on July 12, 2021), and follow-up period (divided into 3-month subperiods). In a sensitivity analysis, we excluded individuals from the unvaccinated group who were vaccinated during the follow-up period.

Additionally, we assessed whether the results might have been influenced by unmeasured confounding by testing whether the occurrence of 2 negative control outcomes (NCOs), namely hospitalization for traumatic injury (*ICD-10* codes beginning with S) and hospitalization for unintentional injury (*ICD-10* codes X0-X59, Y86-Y86, and those beginning with V or W), differed between vaccinated and unvaccinated individuals. These NCOs, which have been used in other pharmaco-epidemiological studies,^33^ were selected because they are unlikely to be directly related to vaccination status and because they occurred frequently enough in our population. We thus aimed to capture residual confounding factors, such as differential risk behaviors associated with vaccination status. We provide calibrated HRs with 95% CIs. We also calculated the E value, quantifying the minimum association strength required for an unmeasured confounding factor to explain the observed association. We also compared the primary causes of death, categorized by major categories of the *ICD-10* classification, between vaccinated and unvaccinated individuals (eTable 1 in Supplement 1).

All calculations were performed using SAS Enterprise Guide version 8.3 software (SAS Institute). Figures and bootstrap confidence intervals were generated using R software version 4.1.2 (R Project for Statistical Computing). HRs were considered statistically significant if the 95% CI did not cross the null.

## Results

### Four-Year All-Cause Mortality

Our cohort included 22 767 546 individuals vaccinated with a first dose of mRNA vaccine between May 1 and October 31, 2021, and 5 932 443 individuals still unvaccinated by November 1, 2021 (eFigure 2 in Supplement 1). Compared with the unvaccinated group, vaccinated individuals were older (mean [SD] age 37.1 [11.4] vs 38.0 [11.8] years), more frequently women (2 876 039 [48.5%] vs 11 688 603 [51.3%]), less deprived (with CSS: 1 240 563 [20.9%] vs 2 087 128 [9.2%]; residence in commune belonging to the most disadvantaged quintile: 1 600 040 [27.0%] vs 4 349 295 [19.1%]) and had more cardiometabolic comorbidities (464 596 [7.8%] vs 2 126 250 [9.3%]). Most vaccinated patients had received a complete primary vaccination (20 920 855 [91.9%]), including 3 441 857 (15.1%) who received a booster dose by the start of the follow-up (Table 1). The distribution of vaccinated and unvaccinated individuals by index date is shown in eFigure 3 in Supplement 1. In the unvaccinated group, 641 910 individuals (10.8%) were vaccinated during follow-up. The main risk factors for all-cause mortality in individuals aged 18 to 59 years were identified, regardless of the vaccination status. Notably, the strongest associations were observed with age, sex, deprivation, addictive consumption of alcohol or tobacco, history of cancer, Down syndrome, and chronic dialysis (eTable 2 in Supplement 1).

During a median follow-up of 45 months, 98 429 (0.4%) and 32 662 (0.6%) deaths occurred in the vaccinated and unvaccinated groups, respectively, including 280 (0.001%) and 308 (0.005%) deaths during hospitalization for COVID-19. Additionally, 17 687 (0.1%) and 13 359 (0.2%) individuals in the vaccinated and unvaccinated groups, respectively, were hospitalized for COVID-19. Median (IQR) follow-up duration was similar in the vaccinated and unvaccinated groups (45.3 [44.1-46.1] months; 44.9 [43.7-46.0] months, respectively). The crude association between COVID-19 vaccination and all-cause mortality was 0.70 (95% CI, 0.70-0.71). After standardizing the characteristics of vaccinated individuals to those of unvaccinated individuals, we observed a 25% lower standardized incidence of all-cause death in vaccinated individuals compared with unvaccinated ones (weighted HR [wHR], 0.75 [95% CI, 0.75-0.76]). A 74% lower risk was observed for hospital mortality due to COVID-19 (wHR, 0.26 [95% CI, 0.21-0.32]), while an estimate equivalent to that of the main analysis was found when hospital deaths due to COVID-19 were excluded (wHR, 0.76 [95% CI, 0.75-0.77]) (results not shown). Results were consistent when stratified by age, sex, region, CSS coverage, social deprivation index, history of COVID-19, and history of chronic disease as well as when excluding individuals in the unvaccinated group who were vaccinated during follow-up (Figure). A stronger association was observed among individuals aged 18 to 29 years, although the underlying reasons remain unclear and warrant further investigation.

**Figure.**
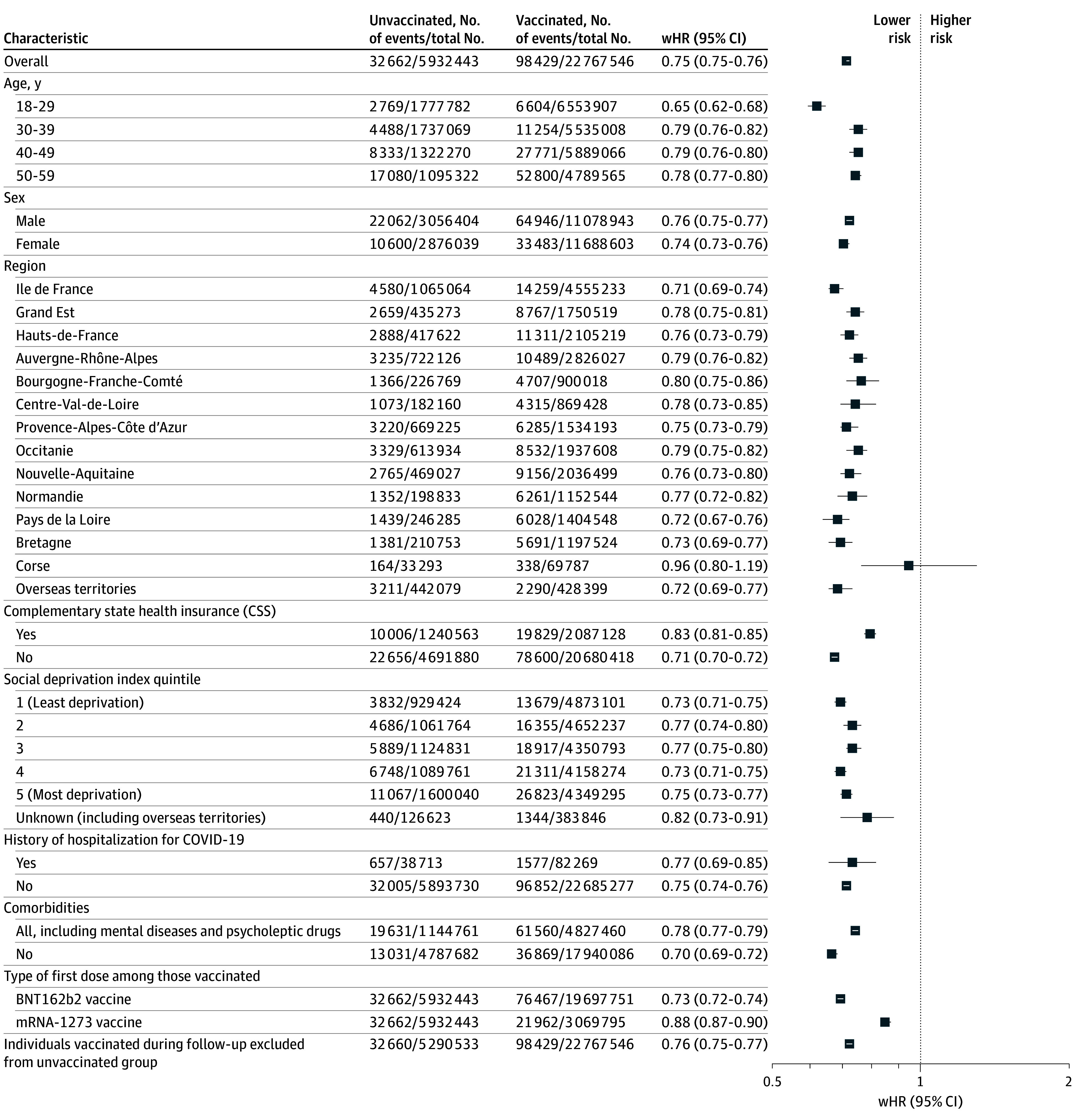
Estimation of All-Cause Mortality at 4 Years in Vaccinated Compared With Unvaccinated Individuals Using Weighted Cox Models: Main and Stratified Analyses Note that individuals are, by definition, alive during the first 6 months of follow-up. wHR indicates weighted hazard ratio.

The characteristics of patients according to the type of first dose received are presented in eTable 3 in Supplement 1. Stratification by the type of first dose received showed, compared with unvaccinated individuals, a 27% reduction in the risk of all-cause mortality in those who received the first dose of the BNT162b2 vaccine (wHR, 0.73 [95% CI, 0.72-0.74]) and a 12% reduction in the risk in those who received the first dose of the mRNA-1273 vaccine (wHR, 0.88 [95% CI, 0.87-0.90]) (Figure).

Patient characteristics were also described by inclusion period, ie, before vs after the announcement of the implementation of the health pass on July 12, 2021, which restricted access to certain public establishments, services, and events in France (eTable 4 in Supplement 1). Stratification by these 2 inclusion subperiods showed a stronger association between death and exposure for those included before vs after July 12, 2021 (wHR, 0.69 [95% CI, 0.67-0.69] vs wHR, 0.93 [95% CI, 0.91-0.95]) (eFigure 4 in Supplement 1). Dividing the follow-up period into 3-month subperiods showed a decrease in the strength of the association over time, starting at 0.61 (95% CI, 0.58-0.64) between 6 and 9 months’ follow-up and asymptotically approaching 0.80 from approximately 15 months, reaching 0.79 (95% CI, 0.75-0.82) between 39 and 42 months’ follow-up (eFigure 5 in Supplement 1).

The E value was 1.99, with a lower confidence interval limit of 1.97. This means that, given the observed negative association between mortality and exposure, the strength of this confounder would need to be 2 with exposure and 0.5 with mortality or vice versa (0.5 with exposure and 2 with mortality) to fully explain the observed association. Calibration of all-cause mortality with the results of NCOs reduced the strength of the association to 0.80 (95% CI, 0.79-0.81) for calibration with traumatic injury and to 0.83 (95% CI, 0.81-0.84) for calibration with involuntary injury, respectively (eFigure 4 in Supplement 1).

The main causes of death were cancer (769 and 853 cases per million in vaccinated and unvaccinated individuals, respectively), external causes of mortality (493 and 597 cases per million, including, among others, unintentional injuries, such as transportation crashes, falls, and drownings, as well as suicides or self-inflicted injuries) and diseases of the circulatory system (282 and 367 cases per million) (Table 2). Vaccinated individuals had a lower risk of death compared with unvaccinated individuals regardless of the cause of death. The proportion of deaths due to COVID-19 among all deaths decreased over the follow-up period, particularly in unvaccinated individuals. Between 6 and 9 months’ follow-up, the proportion of COVID-19–related deaths was 10.5% in the unvaccinated group and 1.9% in the vaccinated group; this proportion was 0.5% and 0.3%, respectively, between 24 and 27 months of follow-up (eFigure 6 in Supplement 1).

**Table 2.** Comparison of Causes of Death Between Vaccinated and Unvaccinated Individuals up to December 31, 2023, Using Weighted Cox Models Among Those Included in the 4-Year Mortality Study^a^

*ICD-10*	Primary causes of death	Incidence per 1 million		Hazard ratio	
		Among vaccinated	Among unvaccinated	Crude	Weighted
NA	Unknown (unlinkable)	199	327	0.55 (0.52-0.58)	0.58 (0.55-0.61)
A, B	Infectious and parasitic diseases	28	45	0.55 (0.48-0.64)	0.63 (0.54-0.73)
C, D0-D4	Tumors	769	853	0.81 (0.79-0.84)	0.85 (0.83-0.88)
C50 and D05	Including breast cancer	76	103	0.67 (0.61-0.73)	0.68 (0.61-0.74)
C10-C20, D010-D012	Including colorectal cancer	62	66	0.85 (0.76-0.95)	0.89 (0.80-0.99)
C33, C34, D021, D022	Including lung cancer	174	194	0.81 (0.76-0.86)	0.85 (0.79-0.90)
Other codes in C or D0 to D04	Including other cancer	456	491	0.84 (0.80-0.87)	0.89 (0.85-0.92)
D5-D8	Diseases of the blood, hematopoietic organs, and certain immune system disorders	6	13	0.46 (0.35-0.60)	0.50 (0.35-0.68)
E	Endocrine, nutritional, and metabolic diseases	51	74	0.62 (0.56-0.70)	0.73 (0.65-0.83)
F	Mental and behavioral disorders	88	135	0.59 (0.54-0.64)	0.65 (0.59-0.70)
G, H	Diseases of the nervous system and sensory organs	66	109	0.55 (0.50-0.60)	0.62 (0.57-0.67)
I	Diseases of the circulatory system	282	367	0.69 (0.66-0.73)	0.76 (0.73-0.79)
J	Diseases of the respiratory system	60	95	0.57 (0.52-0.63)	0.66 (0.59-0.74)
K	Diseases of the digestive system	139	172	0.72 (0.67-0.78)	0.84 (0.80-0.90)
L	Diseases of the skin and subcutaneous tissue	2	5	0.40 (0.26-0.64)	0.49 (0.30-0.75)
M	Diseases of the musculoskeletal system, muscles, and connective tissue	9	11	0.69 (0.52-0.91)	0.85 (0.61-1.13)
N	Diseases of the genitourinary system	9	14	0.54 (0.42-0.70)	0.69 (0.52-0.87)
O	Pregnancy, childbirth, and the puerperium	2	4	0.36 (0.21-0.60)	0.41 (0.22-0.64)
P	Certain conditions originating in the perinatal period	0	1		-
Q	Congenital malformations and chromosomal anomalies	10	18	0.51 (0.41-0.64)	0.59 (0.47-0.78)
R	Symptoms, signs, and abnormal clinical and laboratory findings, not elsewhere classified	276	414	0.60 (0.57-0.63)	0.68 (0.65-0.71)
U071, U072, U109	COVID-19	18	85	0.20 (0.17-0.23)	0.26 (0.22-0.30)
V, W, X, Y	External causes of morbidity and mortality	493	597	0.74 (0.72-0.77)	0.78 (0.75-0.81)
V01-V99	Including transport crashes	65	86	0.68 (0.61-0.75)	0.74 (0.67-0.83)
W00-W19	Including falls	21	26	0.72 (0.60-0.87)	0.80 (0.63-0.97)
W65-W74	Including drownings	9	13	0.62 (0.48-0.80)	0.73 (0.57-0.99)
W20-W64, W75-X59	Including other unintentional injuries	105	134	0.70 (0.65-0.76)	0.80 (0.74-0.87)
X60-X84	Including suicides and self-inflicted injuries	229	222	0.93 (0.88-0.99)	0.88 (0.83-0.94)
Any	Any	2505	3337	0.68 (0.67-0.69)	0.73 (0.72-0.74)

Abbreviations: *ICD-10*, *International Statistical Classification of Diseases and Related Health Problems, Tenth Revision*; NA, not applicable.

^a^ Individuals are, by definition, alive during the first 6 months of follow-up; 8% of deaths could not be linked to cause-of-death data.

### All-Cause Short-Term Mortality Following Vaccination 

In the short-term mortality substudy (May 2021 to July 2022), 60 997 deaths occurred among unvaccinated or first-dose mRNA recipients (5967 individuals excluded for initial non-mRNA vaccination). Main causes of death were cancer (19 598 [32.1%]), external causes (10 412 [17.1%]), and circulatory diseases (6146 [10.1%]) (eTable 5 in Supplement 1). All-cause mortality was lower within 6 months following COVID-19 vaccination, regardless of the dose administered, compared with the control periods (relative incidence [RI], 0.71; 95% CI, 0.69-0.73) (Table 3),^34^ with a stronger negative association for COVID-19 mortality (RI, 0.39; 95% CI, 0.32-0.47). No substantial differences were observed across 3-month subperiods for all-cause or cause-specific mortality (eTable 6 in Supplement 1).

**Table 3.** RI of Short-Term Mortality, All Causes, by Cancer, External Causes, Circulatory Diseases, and COVID-19, Within 6 Months Following Vaccination, Using Adapted SCCS Models^a^

Risk window^b^	Cause of death, RI (95% CI)				
	All-cause	Tumor	Circulatory diseases	External causes	COVID-19
6 mo After dose 1	0.65 (0.63-0.67)	0.71 (0.67-0.76)	0.63 (0.57-0.71)	0.63 (0.58-0.68)	0.73 (0.59-0.91)
6 mo After dose 2	0.76 (0.74-0.79)	0.85 (0.81-0.89)	0.74 (0.66-0.83)	0.78 (0.71-0.86)	0.29 (0.23-0.36)
6 mo After dose 3	0.80 (0.76-0.84)	0.83 (0.77-0.89)	0.76 (0.65-0.88)	0.95 (0.83-1.09)	0.40 (0.30-0.52)
6 mo After any dose	0.71 (0.69-0.73)	0.80 (0.77-0.84)	0.68 (0.62-0.76)	0.67 (0.61-0.72)	0.39 (0.32-0.47)

Abbreviations: RI, relative incidence; SCCS, self-controlled case series.

^a^ The few events occurring on the day of vaccination were considered separately.

^b^ When the RI of the first vaccine dose is estimated, second doses (should they occur) are suppressed, but counts of deaths that occur during such second-dose exposure periods are adjusted to account for the fact that second doses were suppressed. To estimate the vaccination effect (RI), a pseudo-likelihood approach is implemented in R. This is done by replacing the event count n with n/RI2, where RI2 is the RI of the second dose. Second doses are suppressed when RIs of the first dose are estimated because the presence and timing of such second doses may be affected by the event occurring after the first dose.^34^

## Discussion

To our knowledge, this is the first national population-based study to examine differences in all-cause mortality between individuals who did and did not receive COVID-19 vaccines 4 years after their first dose of COVID-19 vaccination. We estimated a 25% lower risk of all-cause mortality in vaccinated compared with unvaccinated adults aged 18 to 59 years. Consistent results were found when stratifying by demographic and socioeconomic variables, history of COVID-19, type of first dose of mRNA vaccine, history of chronic disease, and time periods as well as when excluding individuals from the unvaccinated group who got vaccinated during follow-up. Although calibration on NCOs reduced the strength of the estimated association, an approximate 20% reduction in 4-year mortality remained in the vaccinated group. In line with the literature, we also observed lower short-term mortality in vaccinated individuals, with a 29% reduction within 6 months following COVID-19 vaccination.

Assessing long-term all-cause mortality associated with vaccination presented several methodological challenges. First, individuals who choose vaccination may differ from those who do not, potentially introducing confounding bias. This could be due to a healthy-vaccinee effect,^35^ where healthier individuals opt for vaccination, or a frailty-related bias, where those in poorer health may avoid it.^36^ These differences were at least partly addressed using inverse probability of treatment weighting Cox models that account for many demographic characteristics and health conditions as well as socioeconomic status, which influence both mortality and vaccination propensity, but also by NCOs, which should detect and correct for residual confounding,^37^ as long as they are influenced by the same unmeasured confounders but not causally impacted by the exposure. Although vaccinated individuals in our study were generally older and tended to have more comorbidities—factors that would typically bias the association toward higher mortality in the vaccinated group—this was not observed in the crude hazard ratio. We also found that vaccinated individuals were more socioeconomically advantaged and likely benefited from better health care management, variables insufficiently captured in our data. These factors may partly explain the observed negative association between vaccination and mortality, counterbalancing the effects of age and comorbidities.

Second, the dynamics of vaccination posed a challenge in considering time to event for the respective exposure groups to avoid immortal time bias.^38^ To study long-term mortality in the unvaccinated group and prevent significant loss to follow-up due to individuals rapidly getting vaccinated, we included individuals who remained unvaccinated (and alive) 3 months after the peak of mass vaccination on November 1, 2021. Starting follow-up from this date would have resulted in unequal follow-up durations across groups. Therefore, we introduced a random index date for the unvaccinated group based on the vaccinated individuals’ first injection dates. As unvaccinated individuals were alive between their random index date and November 1, 2021, corresponding to a maximum duration of 6 months, starting follow-up from the index date would have introduced immortal time bias. Therefore, we initiated the study of long-term mortality 6 months after the index date, while mortality within 6 months after vaccination was analyzed separately using SCCS models. While the SCCS models are well-suited for short-term vaccine safety studies, they are less appropriate for long-term mortality, particularly due to limited control periods among vaccinated individuals, and age differences within the same individual across risk or control periods, as age is by far a major risk factor for death. For both analyses, we introduced the calendar period as an adjusting factor to account for the infection dynamics, baseline mortality trends, and the varying propension to get vaccinated.

We did not differentiate between multiple vaccine doses. Our objective was to evaluate the impact of vaccination status from the perspective of public health decision-makers evaluating intervention strategies. In this regard, we focused on estimating the overall association of being vaccinated with mortality, rather than isolating the specific contribution of each additional dose, which represents a distinct research question.

It seems reasonable to assume that by early November 2021, 3 months after the introduction of the mandatory health pass^39^ (delivered when fulfilling one of these conditions: a negative COVID-19 test result, proof of COVID-19 vaccination, or a certificate of recovery from a COVID-19 infection) to enter and exit France as well as to access restaurants, theaters, and nonurgent hospital consultations, the majority of unvaccinated individuals were reluctant to get vaccinated.^40^ A study aimed at characterizing patient hesitancy toward COVID-19 vaccination showed that categorical refusal of vaccination was associated with prior noncompliance with vaccination recommendations, a lower educational level, and a less severe perception of COVID-19.^41^ Moreover, social inequalities in vaccination uptake have been observed in France, despite the availability of free vaccination,^42^ suggesting that socioeconomic status likely influences vaccination adherence. These factors, which cannot be completely accounted for in our database, may be likely responsible for a part of residual unmeasured confounding, as they are associated with both mortality and exposure. The use of NCOs typically allows for at least partial adjustment for these factors. In practice, while the magnitude of the associations has been reduced, a strong negative association persists after their application.

The analysis by cause of death reflects the long-term effectiveness of vaccination on the risk of death from COVID-19, and more broadly on the risk of developing a severe, hospitalized form of COVID-19. This result is consistent with that of a previous study,^43^ which reported a 52% effectiveness of primary vaccination (mostly with an mRNA vaccine) against the risk of hospitalization for COVID-19 more than 9 months after the injection. The proportion of COVID-19 deaths among all deaths decreased over the follow-up period, particularly among unvaccinated individuals, likely reflecting both vaccine-induced protection and changes in viral circulation. The stronger negative association observed between vaccination and all-cause mortality at the first months of the follow-up period may be partly attributable to the higher COVID-19–related mortality in unvaccinated individuals.

Long COVID is a frequently disabling condition that may occur in 10% of individuals with SARS-CoV-2 infections, manifesting with more than 200 symptoms affecting multiple organ systems,^44,45^ with long-term consequences for some individuals, including cardiovascular effects.^46^ Individuals with postacute sequelae of SARS-CoV-2 may have an increased risk of mortality at 1 year,^47^ and the burden related to mortality and health loss may persist during the third year following SARS-CoV-2 infection among hospitalized individuals.^48^ Vaccination tends to reduce the occurrence of these complications,^22^ which may partly explain the strong negative association observed between vaccination and non–COVID-19 mortality.^49^

### Limitations

Our study has several limitations. It has been acknowledged that fraudulent health passes were issued, with an estimated 300 000 fraudulent passes in 2022,^50^ which may introduce classification bias but most likely in a limited manner, although the exact extent of the phenomenon remains uncertain. Second, apart from deaths occurring in hospital following admission for COVID-19 infection, we did not have access to cause-of-death data for the entire follow-up period, but we were able to identify causes for those occurring in the first half of the follow-up, representing 59% of the deaths. However, the distribution of major causes was consistent with national figures for a similar age group.

## Conclusions

In this nationwide cohort study of 28 million individuals, no increased risk of all-cause mortality was observed at 4 years among those vaccinated with COVID-19 mRNA vaccines. While the consistent negative association, even after extensive adjustment and calibration, suggests that residual confounding may persist, a causal link between mRNA vaccination and excess long-term mortality appears highly unlikely. These findings support the long-term safety of BNT162b2 and mRNA-1273 vaccines.

## Notes

Supplement 1. **eMethods 1.** Vaccination Timeline and Eligibility Criteria in France **eMethods 2.** Target Trial Specification and Emulation **eMethods 3.** Estimation of All-Cause Mortality at 6 Months **eFigure 1.** Absolute Standardized Differences Before and After Weighting **eFigure 2.** Flow Chart **eFigure 3.** Weekly Distribution of Index Dates for Vaccinated and Unvaccinated Individuals in the 4-Year Mortality Study **eFigure 4.** Estimation of All-Cause Mortality in Vaccinated vs Unvaccinated Individuals Using Weighted Cox Models With and Without Calibration by Negative Control Outcomes in the 4-year Mortality Study: Main and Stratified Analyses **eFigure 5.** Estimation of All-Cause Mortality in Vaccinated vs Unvaccinated Individuals Using Weighted Cox Models, Dividing the Follow-Up Period Into 3-Month Subperiods, in the 4-Year Mortality Study **eFigure 6.** Proportion of Deaths Attributed to COVID-19 Among All Deaths in Vaccinated and Unvaccinated Individuals by 3-Month Follow-Up Subperiods in the 4-Year Mortality Study **eTable 1.** Primary Causes of Death, Categorized by Major Categories of the ICD-10 Classification **eTable 2.** Risk Factors for All-Cause 4-Year Mortality Estimated From an Adjusted Cox Model **eTable 3.** Characteristics at Baseline of COVID-19 Vaccinated Individuals Included in the 4-Year Mortality Study by Type of First Injection Received **eTable 4.** Characteristics at Baseline of Individuals Included in the 4-Year Mortality Study, Dividing the Inclusion Period Into 2 Periods, ie, Before and After the Announcement of the Implementation of the Vaccine Pass on July 12, 2021 **eTable 5.** Number of Deceased Patients by Vaccination Status in the Study of Short-Term Mortality **eTable 6.** Relative Incidence of Short-Term Mortality, All Causes, by Cancer, External Causes, Circulatory Diseases, and COVID-19, Within 2 subperiods of 3 Months Following Vaccination, Using Adapted SCCS Models

Supplement 2.
Data Sharing Statement
