# Supplementary material for: COVID-19 mRNA Vaccination and 4-Year All-Cause Mortality Among Adults Aged 18 to 59 Years in France
Source: JAMA Netw Open. 2025 Dec 4;8(12):e2546822. doi: 10.1001/jamanetworkopen.2025.46822 (PMC12679329; doi:10.1001/jamanetworkopen.2025.46822)
Supplement: Supplement 1. — eMethods 1. Vaccination Timeline and Eligibility Criteria in France eMethods 2. Target Trial Specification and Emulation eMethods 3. Estimation of All-Cause Mortality at 6 Months eFigure 1. Absolute Standardized Differences Before and After Weighting eFigure 2. Flow Chart eFigure 3. Weekly Distribution of Index Dates for Vaccinated and Unvaccinated Individuals in the 4-Year Mortality Study eFigure 4. Estimation of All-Cause Mortality in Vaccinated vs Unvaccinated Individuals Using Weighted Cox Models With and Without Calibration by Negative Control Outcomes in the 4-year Mortality Study: Main and Stratified Analyses eFigure 5. Estimation of All-Cause Mortality in Vaccinated vs Unvaccinated Individuals Using Weighted Cox Models, Dividing the Follow-Up Period Into 3-Month Subperiods, in the 4-Year Mortality Study eFigure 6. Proportion of Deaths Attributed to COVID-19 Among All Deaths in Vaccinated and Unvaccinated Individuals by 3-Month Follow-Up Subperiods in the 4-Year Mortality Study eTable 1. Primary Causes of Death, Categorized by Major Categories of the ICD-10 Classification eTable 2. Risk Factors for All-Cause 4-Year Mortality Estimated From an Adjusted Cox Model eTable 3. Characteristics at Baseline of COVID-19 Vaccinated Individuals Included in the 4-Year Mortality Study by Type of First Injection Received eTable 4. Characteristics at Baseline of Individuals Included in the 4-Year Mortality Study, Dividing the Inclusion Period Into 2 Periods, ie, Before and After the Announcement of the Implementation of the Vaccine Pass on July 12, 2021 eTable 5. Number of Deceased Patients by Vaccination Status in the Study of Short-Term Mortality eTable 6. Relative Incidence of Short-Term Mortality, All Causes, by Cancer, External Causes, Circulatory Diseases, and COVID-19, Within 2 subperiods of 3 Months Following Vaccination, Using Adapted SCCS Models [file jamanetwopen-e2546822-s001.pdf]

Supplemental Online Content

Semenzato L, Le Vu S, Botton J, et al. COVID-19 mRNA vaccination and 4-year all-cause mortality among adults aged 18 to 59 years in France. *JAMA Netw Open*. 2025;8(12):e2546822. doi:10.1001/jamanetworkopen.2025.46822

eMethods 1. Vaccination Timeline and Eligibility Criteria in France

eMethods 2. Target Trial Specification and Emulation

eMethods 3. Estimation of All-Cause Mortality at 6 Months

eFigure 1. Absolute Standardized Differences Before and After Weighting

eFigure 2. Flow Chart

eFigure 3. Weekly Distribution of Index Dates for Vaccinated and Unvaccinated Individuals in the 4-Year Mortality Study

eFigure 4. Estimation of All-Cause Mortality in Vaccinated vs Unvaccinated Individuals Using Weighted Cox Models With and Without Calibration by Negative Control Outcomes in the 4-year Mortality Study: Main and Stratified Analyses

eFigure 5. Estimation of All-Cause Mortality in Vaccinated vs Unvaccinated Individuals Using Weighted Cox Models, Dividing the Follow-Up Period Into 3-Month Subperiods, in the 4-Year Mortality Study

eFigure 6. Proportion of Deaths Attributed to COVID-19 Among All Deaths in Vaccinated and Unvaccinated Individuals by 3-Month Follow-Up Subperiods in the 4-Year Mortality Study

eTable 1. Primary Causes of Death, Categorized by Major Categories of the ICD-10 Classification

eTable 2. Risk Factors for All-Cause 4-Year Mortality Estimated From an Adjusted Cox Model

eTable 3. Characteristics at Baseline of COVID-19 Vaccinated Individuals Included in the 4-Year Mortality Study by Type of First Injection Received

eTable 4. Characteristics at Baseline of Individuals Included in the 4-Year Mortality Study, Dividing the Inclusion Period Into 2 Periods, ie, Before and After the Announcement of the Implementation of the Vaccine Pass on July 12, 2021

eTable 5. Number of Deceased Patients by Vaccination Status in the Study of Short-Term Mortality

eTable 6. Relative Incidence of Short-Term Mortality, All Causes, by Cancer, External Causes, Circulatory Diseases, and COVID-19, Within 2 subperiods of 3 Months Following Vaccination, Using Adapted SCCS Models

This supplemental material has been provided by the authors to give readers additional information about their work.

© 2025 Semenzato L et al. *JAMA Network Open*.

eMethods 1. Vaccination Timeline and Eligibility Criteria in France

The COVID-19 vaccination campaign began in France on December 27, 2020, primarily using mRNA vaccines, namely the BNT162b2 vaccine (Pfizer-BioNTech©) and the mRNA-1273 vaccine (Moderna©), with the Pfizer-BioNTech© BNT162b2 mRNA vaccine being by far the most widely used. Two adenovirus-based vaccines were also available for primary COVID-19 vaccination, but their use was rapidly restricted, leading to their withdrawal.

Vaccination priority shifted over time based on vaccine availability, initially targeting healthcare workers, nursing home residents, older adults (75+), and individuals with severe or multiple chronic conditions, before expanding to all adults by mid-May 2021. The peak of vaccination uptake was between mid-May and mid-August 2021 and, by November 1, 2021, more than 75% of the population had received at least a first dose of vaccine.

The original Alpha variant caused two COVID-19 waves in France in 2020, followed by Beta and Gamma variants in early 2021 and the Delta variant in mid-2021[1]. Due to waning vaccine effectiveness[2], emerging variants, and relaxed preventive measures, severe COVID-19 cases increased among vaccinated individuals at the end of 2021. This led authorities to recommend a first mRNA booster dose from November 2021, extended to all adults, and a second booster dose from March 2022[3], initially for older adults and later for at-risk individuals[4,5].

1. Santé Publique France. Coronavirus : circulation des variants du SARS-CoV-2. https://www.santepubliquefrance.fr/dossiers/coronavirus-covid-19/coronavirus-circulation-des-variants-du-sars-cov-2.
2. Thomas, S. J. & Moreira, E. D. Safety and Efficacy of the BNT162b2 mRNA Covid-19 Vaccine through 6 Months. *n engl j med* 13 (2021).
3. Conseil d'Orientation de la Stratégie Vaccinale. *Addendum du 18 février 2022 à l'avis du 19 janvier 2022 Opportunité de la mise en place d'une seconde dose de rappel vaccinal*.

© 2025 Semenzato L et al. *JAMA Network Open*.

4. Gouvernement. Les personnes de 60 ans et plus peuvent effectuer une deuxième dose de rappel contre le Covid-19.
5. Avis n°2022.0043/AC/SESPEV du 13 juillet 2022 du Collège de la Haute Autorité de santé relatif à la place d’une dose de rappel additionnelle des vaccins contre la Covid-19 dans la stratégie vaccinale.

© 2025 Semenzato L et al. *JAMA Network Open*.

eMethods 2. Target Trial Specification and Emulation

Design	Target trial specification	Target trial emulation
Eligibility criteria	All alive individuals aged 18 to 59 years and residing in France as of November 1, 2021	All alive individuals aged 18 to 59 years, residing in France as of November 1, 2021 and having received any healthcare reimbursement in 2020 (to limit loss of follow-up)
Inclusion period	Between May 1 and October 31, 2021	Between May 1 and October 31, 2021
Exclusion criteria	- Individuals vaccinated before May 1, 2021 or who received a first dose of another (i.e. non-mRNA based) COVID-19 vaccine during the inclusion period	- Individuals vaccinated before May 1, 2021 or who received a first dose of another (i.e. non-mRNA based) COVID-19 vaccine during the inclusion period<br>- Individuals deceased or vaccinated (for the unexposed group) during the 6-month grace period between the index date and the effective start of follow-up
Treatment strategies	- Exposure to a first dose of COVID-19 mRNA vaccine<br>- Unexposure to COVID-19 vaccine	- Exposure to COVID-19 vaccination was defined as the administration of a first dose of an mRNA vaccine<br>- The unvaccinated group includes all individuals who had not received a COVID-19 vaccine as of November 1, 2021
Treatment assignment	Eligible individuals would be randomly assigned to their treatment strategy	While the index date for vaccinated individuals corresponds to the date of their first vaccine dose, unvaccinated individuals lack a comparable index date. An index date was randomly assigned to each unvaccinated individual by drawing into the distribution of first vaccination dates observed among vaccinated individuals.
Time zero	After treatment assignment	Follow-up for all individuals, whether vaccinated or not, began 6 months after the index date (time zero). This 6-month period was analyzed separately using a dedicated methodology
Outcome	All-cause death and main-cause death	All-cause death and main-cause death
Follow-up	Time to event was censored at all-cause death, administration of a COVID-19 vaccine for unexposed individuals, or study termination on March 31, 2025, whichever occurred first.	Time to event was censored at all-cause death, administration of a COVID-19 vaccine for unexposed individuals, or study termination on March 31, 2025, whichever occurred first.
Data analysis plan	Cox models	Weighted Cox models

© 2025 Semenzato L et al. *JAMA Network Open*.

eMethods 3. Estimation of All-Cause Mortality at 6 Months

Population

We used a similar methodology as in Botton et al.[1] and Jabagi et al.[2] to estimate the potential risk of all-cause mortality during the first 6 months following vaccination[3].

Observation period extended from May 1, 2021, start of mass vaccination for adults, until July 31, 2022. Eligible participants were all individuals deceased during the observation period and aged between 18 and 59 at the time of death.

All case patients (individuals deceased) who received at least one injection of an mRNA COVID-19 vaccine and all unvaccinated case patients (until July 30, 2022) were included. The inclusion of unvaccinated persons contributed directly to the assessment of baseline temporal effects and indirectly to the estimation of associations between vaccination and mortality because the occurrence of the event prevents the vaccination.

Exposure periods

The exposure period was defined as the 6 months after each of the first, second, third and fourth doses of the vaccines (when the number of events allowed) to obtain relative incidence (RI) estimates, considering the day of vaccination (day 0) separately. We also calculated RI estimates by subdividing the exposure period into 2 sub-periods of 3 months. All other observation times were considered as baseline periods.

Statistical analysis

The standard SCCS method has been adapted as described in previous studies[1–3].

The RIs were calculated for deaths occurring during each exposure sub-period and over the entire exposure period compared with the non-exposed period. The RI estimates were adjusted for temporality (in 7-day increments) to account for any temporal change in background rates of both vaccination and mortality. RIs were also adjusted for age by stratifying the models by age group: 18-

© 2025 Semenzato L et al. *JAMA Network Open*.

29, 30-39, 40-44, 45-49, and by each year from 50 to 59 years. Then, for each sub-period and for the entire exposure period, the overall estimate for individuals aged 18 to 59 year-olds was calculated by averaging the beta values for each age group, and weighting them by the inverse of their variance. The numbers of case patients who received a fifth dose over the observation period were insufficient to obtain reliable RI estimates for this dose. The same was true for some analyses of the fourth dose. Thus, the RI estimates refer to the first, second, and third doses. The relative incidences of the three main causes of death, i.e. cancer, external causes of mortality and circulatory diseases, as well as COVID-19 mortality, were also calculated.

All calculations were performed using R software, version 4.1.2. with package SCCS version 1.6.

References

1. Botton, J. *et al.* Risk for Myocardial Infarction, Stroke, and Pulmonary Embolism Following COVID-19 Vaccines in Adults Younger Than 75 Years in France. *Ann Intern Med* **175**, 1250–1257 (2022).
2. Jabagi, M. J. *et al.* Myocardial Infarction, Stroke, and Pulmonary Embolism After BNT162b2 mRNA COVID-19 Vaccine in People Aged 75 Years or Older. *JAMA* **327**, 80 (2022).
3. Ghebremichael-Weldeselassie, Y. *et al.* A modified self-controlled case series method for event-dependent exposures and high event-related mortality, with application to COVID-19 vaccine safety. *Statistics in Medicine* **41**, 1735–1750 (2022).

© 2025 Semenzato L et al. *JAMA Network Open*.

eFigure 1. Absolute Standardized Differences Before and After Weighting

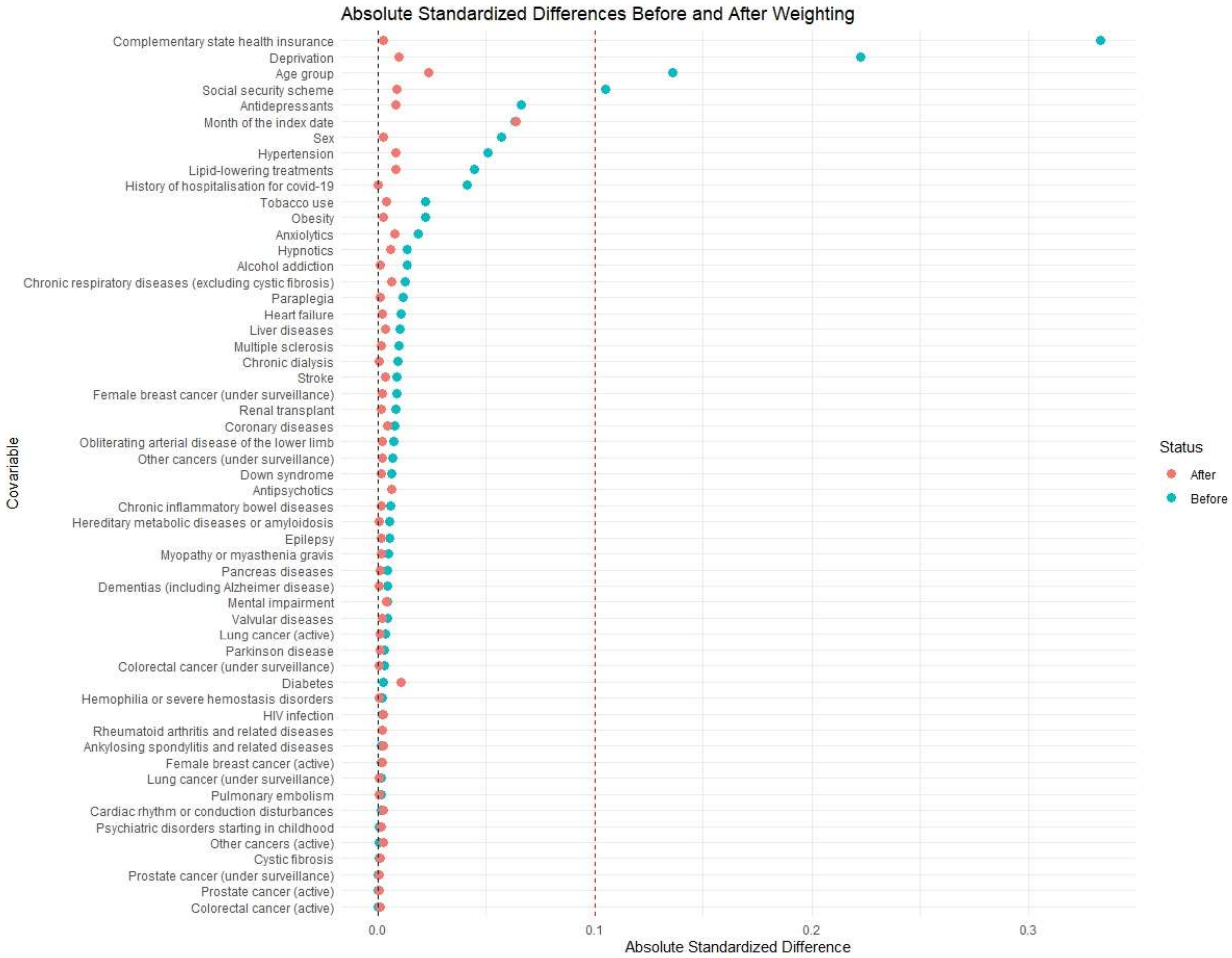

© 2025 Semenzato L et al. *JAMA Network Open*.

eFigure 2. Flow Chart

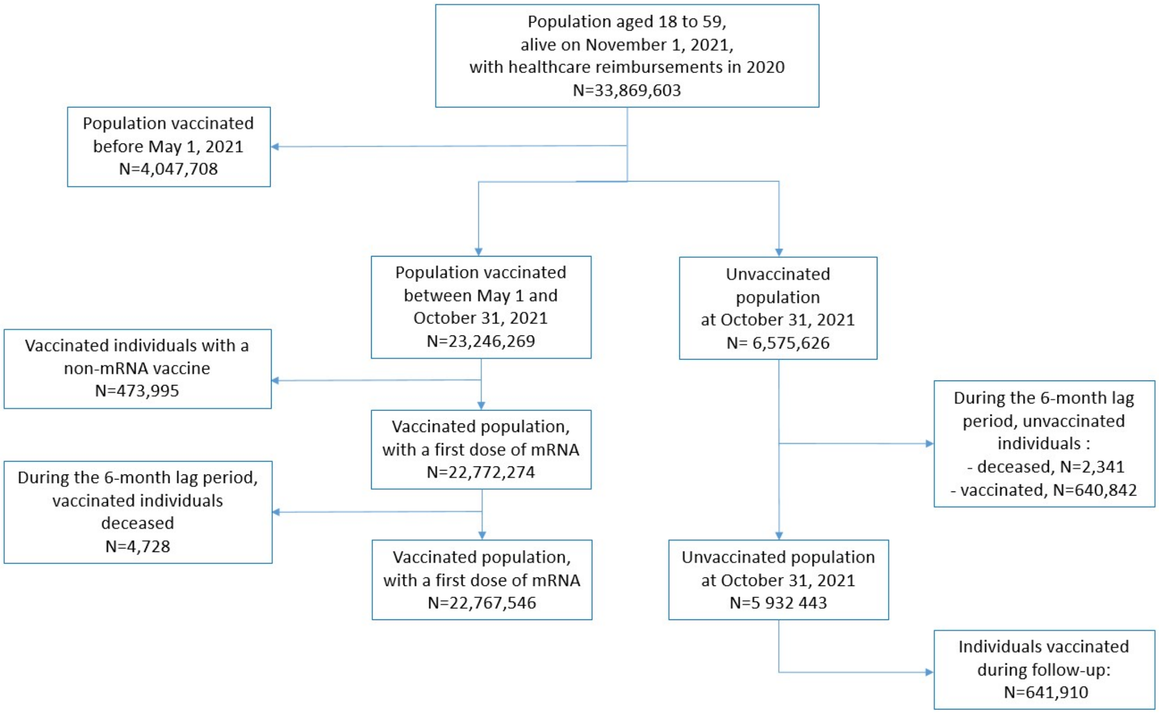

eFigure 3. Weekly Distribution of Index Dates for Vaccinated and Unvaccinated Individuals in the 4-Year Mortality Study

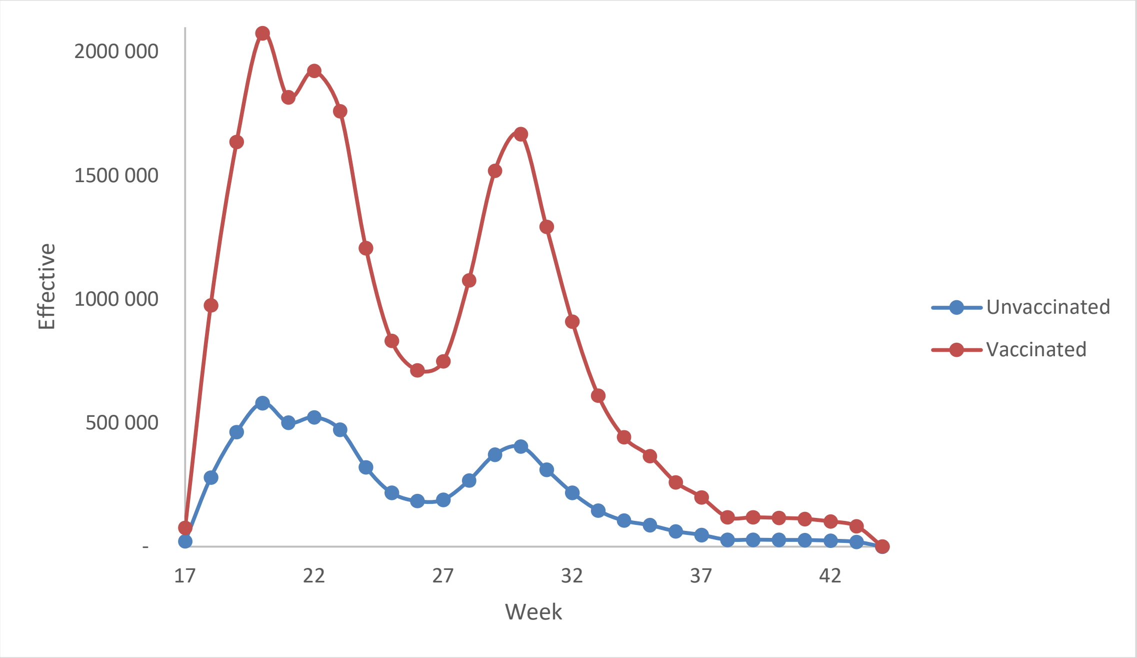

© 2025 Semenzato L et al. *JAMA Network Open*.

eFigure 4. Estimation of All-Cause Mortality in Vaccinated vs Unvaccinated Individuals Using Weighted Cox Models With and Without Calibration by Negative Control Outcomes in the 4-year Mortality Study: Main and Stratified Analyses

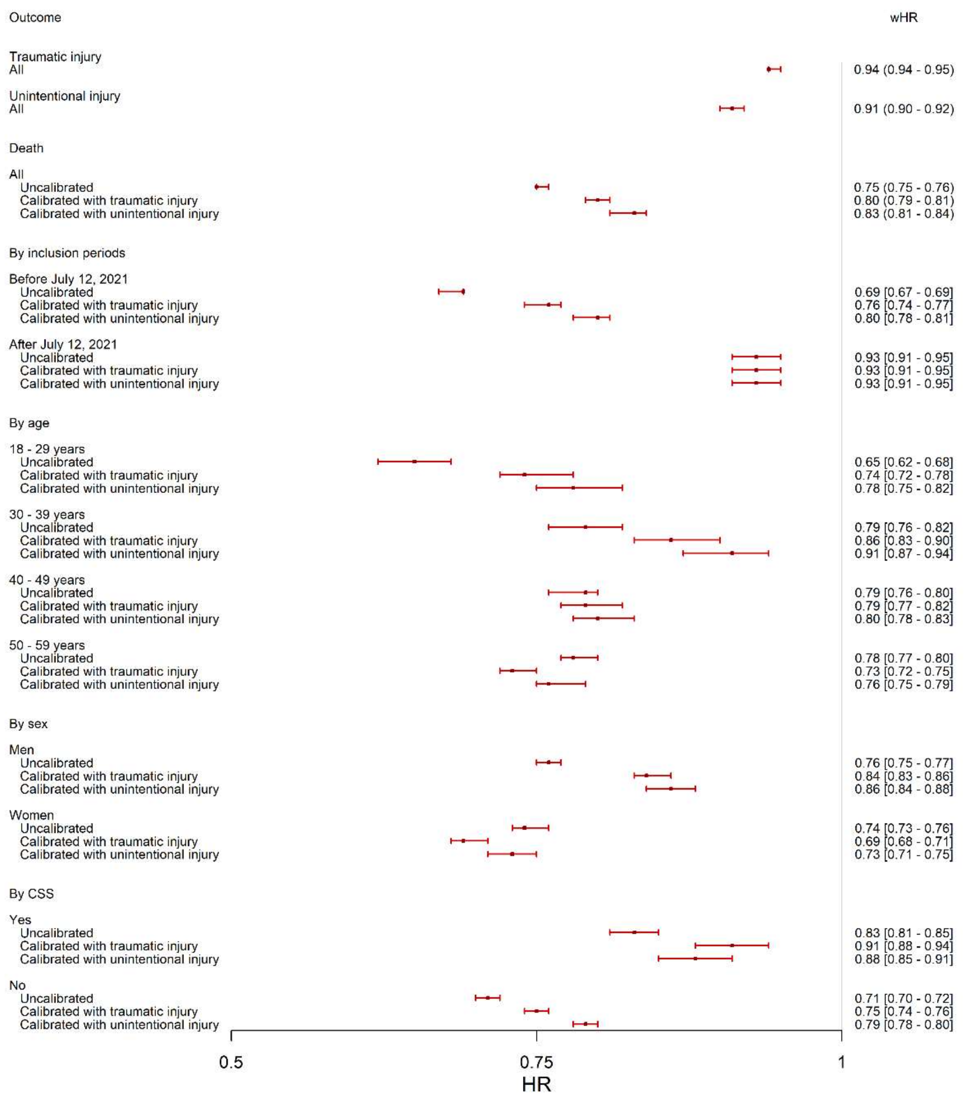

*Note that individuals are, by definition, alive during the first 6 months of follow-up.

© 2025 Semenzato L et al. *JAMA Network Open*.

eFigure 5. Estimation of All-Cause Mortality in Vaccinated vs Unvaccinated Individuals Using Weighted Cox Models, Dividing the Follow-Up Period Into 3-Month Subperiods, in the 4-Year Mortality Study

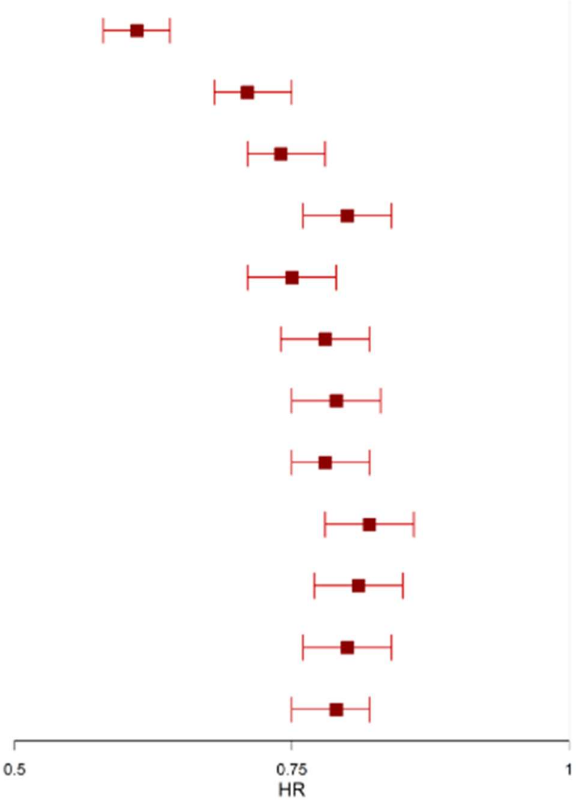

Outcome	Unvaccinated Events/Number	Vaccinated Events/Number	wHR
3-months follow-up periods			
6 - 9 months	2,990/5,932,443	6,638/22,767,546	0.61 (0.58 - 0.64)
9 - 12 months	2,391/5,400,061	6,668/22,760,908	0.71 (0.68 - 0.75)
12 - 15 months	2,412/5,343,225	6,961/22,754,240	0.74 (0.71 - 0.78)
15 - 18 months	2,382/5,314,601	7,469/22,747,279	0.80 (0.76 - 0.84)
18 - 21 months	2,433/5,297,091	7,240/22,739,810	0.75 (0.71 - 0.79)
21 - 24 months	2,303/5,284,760	7,238/22,732,570	0.78 (0.74 - 0.82)
24 - 27 months	2,371/5,279,391	7,357/22,725,332	0.79 (0.75 - 0.83)
27 - 30 months	2,482/5,276,110	7,906/22,717,975	0.78 (0.75 - 0.82)
30 - 33 months	2,472/5,271,776	8,152/22,710,069	0.82 (0.78 - 0.86)
33 - 36 months	2,436/5,268,540	7,952/22,701,917	0.81 (0.77 - 0.85)
36 - 39 months	2,501/5,265,919	8,016/22,693,965	0.80 (0.76 - 0.84)
39 - 42 months	2,654/5,263,362	8,380/22,685,949	0.79 (0.75 - 0.82)

*Note that individuals are, by definition, alive during the first 6 months of follow-up. 8,452 and 2,835 deaths in vaccinated and unvaccinated individuals respectively are missing from this graph, as they occurred beyond 42 months' follow-up.

© 2025 Semenzato L et al. *JAMA Network Open.*

eFigure 6. Proportion of Deaths Attributed to COVID-19 Among All Deaths in Vaccinated and Unvaccinated Individuals by 3-Month Follow-Up Subperiods in the 4-Year Mortality Study

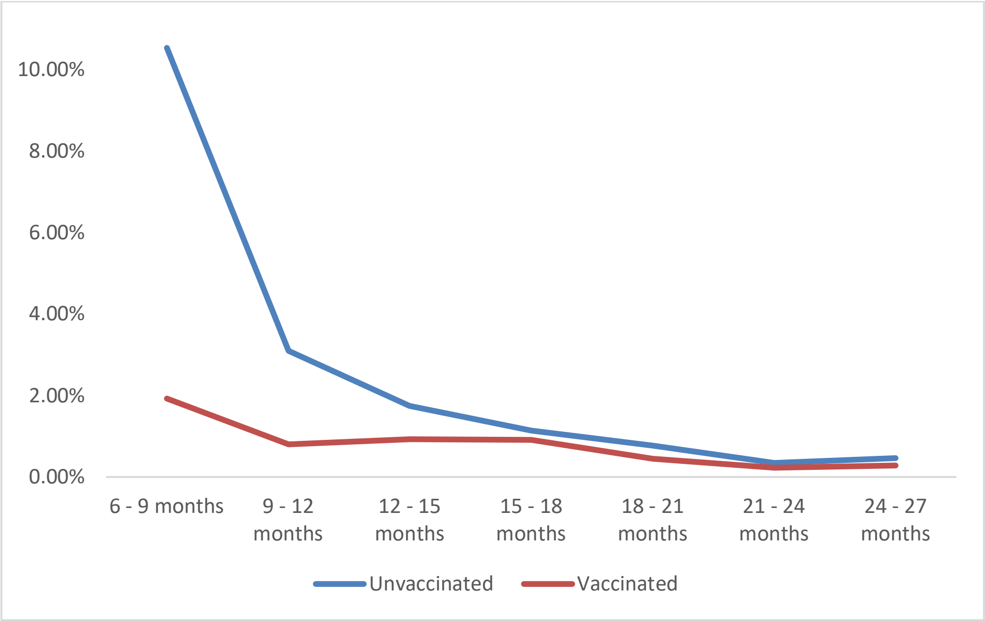

© 2025 Semenzato L et al. *JAMA Network Open*.

eTable 1. Primary Causes of Death, Categorized by Major Categories of the *ICD-10* Classification

ICD-10	Primary causes of death
	Unknown (unlinkable)
A,B	Infectious and parasitic diseases
C,D0-D4	Tumors
C50,D05	including breast cancer
C10-C20,D010,D011,D012	including colorectal cancer
C33,C34,D021,D022	including lung cancer
Other codes in C or D0-D04	including other cancer
D5-D8	Diseases of the blood, hematopoietic organs, and certain immune system disorders
E	Endocrine, nutritional, and metabolic diseases
F	Mental and behavioral disorders
G,H	Diseases of the nervous system and sensory organs
I	Diseases of the circulatory system
J	Diseases of the respiratory system
K	Diseases of the digestive system
L	Diseases of the skin and subcutaneous tissue
M	Diseases of the musculoskeletal system, muscles, and connective tissue
N	Diseases of the genitourinary system
O	Pregnancy, childbirth, and the puerperium
P	Certain conditions originating in the perinatal period
Q	Congenital malformations and chromosomal anomalies
R	Symptoms, signs, and abnormal clinical and laboratory findings, not elsewhere classified
U071,U072,U109	COVID-19
V,W,X,Y	External causes of morbidity and mortality
V01-V99	including transport accidents
W00-W19	including accidental falls
W65-W74	including drownings
W20-W64,W75-X59	including other accidents
X60-X84	including suicides and self-inflicted injuries

© 2025 Semenzato L et al. *JAMA Network Open*.

eTable 2. Risk Factors for All-Cause 4-Year Mortality Estimated From an Adjusted Cox Model

	All				
	Deceased		Alive		Adjusted Hazard Ratio
**Characteristics**	**131 091**	**%**	**28 568 898**	**%**	
**Age (year) - mean (std)**	47.6 (9.8)		37.8 (11.7)		
**Age**					
18 - 29	9 365	7,1%	8 322 324	29,1%	0.58 (0.56 - 0.59)
30 - 39	15 742	12,0%	7 256 335	25,4%	1
40 - 49	36 104	27,5%	7 175 232	25,1%	2.01 (1.97 - 2.04)
50 - 59	69 880	53,3%	5 815 007	20,4%	3.89 (3.82 - 3.96)
**Sex**					
Male	87 008	66,4%	14 048 339	49,2%	2.00 (1.97 - 2.02)
Female	44 083	33,6%	14 520 559	50,8%	1
**Complementary state health insurance (CSS)**	29 835	22,8%	3 297 856	11,5%	1.49 (1.47 - 1.51)
**Social deprivation index (quintiles)**					
1 (the least deprivation)	17 511	13,4%	5 785 014	20,2%	1
2	21 041	16,1%	5 692 960	19,9%	1.07 (1.04 - 1.09)
3	24 806	18,9%	5 450 818	19,1%	1.17 (1.14 - 1.19)
4	28 059	21,4%	5 219 976	18,3%	1.27 (1.24 - 1.29)
5 (the most deprivation)	37 890	28,9%	5 911 445	20,7%	1.37 (1.34 - 1.39)
Unknown	1 784	1,4%	508 685	1,8%	1.17 (1.11 - 1.24)
**Covid-19 vaccination exposure on November 1, 2021**					
Unvaccinated	32 662	24,9%	5 899 781	20,7%	1
Vaccinated	98 429	75,1%	22 669 117	79,3%	0.71 (0.70 - 0.72)
**Lifestyle habit**					
Alcohol addiction	20 121	15,3%	377 940	1,3%	3.59 (3.52 - 3.66)
Tobacco use	19 724	15,0%	1 392 449	4,9%	1.27 (1.25 - 1.29)
**Comorbidities**					
**Cardiometabolics**	39 171	29,9%	2 551 675	8,9%	
Obesity	1 705	1,3%	232 653	0,8%	1.11 (1.06 - 1.17)
Diabetes	11 183	8,5%	554 426	1,9%	1.51 (1.48 - 1.55)
Lipid-lowering treatments	12 552	9,6%	631 805	2,2%	0.90 (0.88 - 0.92)
Hereditary metabolic diseases or amyloidosis	430	0,3%	32 051	0,1%	1.57 (1.43 - 1.73)
Hypertension	26 596	20,3%	1 597 764	5,6%	1.39 (1.37 - 1.41)
Coronary diseases	5 298	4,0%	159 939	0,6%	1.31 (1.27 - 1.35)
Obliterating arterial disease of the lower limb	2 973	2,3%	43 788	0,2%	1.98 (1.91 - 2.06)
Cardiac rhythm or conduction disturbances	2 678	2,0%	103 903	0,4%	1.28 (1.23 - 1.33)
Heart failure	2 575	2,0%	29 500	0,1%	2.08 (1.99 - 2.18)
Valvular diseases	1 035	0,8%	26 604	0,1%	1.60 (1.50 - 1.71)
Stroke	3 164	2,4%	102 481	0,4%	1.62 (1.56 - 1.68)
**Respiratory diseases**					
Chronic respiratory diseases (excluding cystic fibrosis)	12 407	9,5%	923 669	3,2%	1.36 (1.34 - 1.39)
Cystic fibrosis	46	0,0%	2 240	0,0%	4.76 (3.57 - 6.36)
Pulmonary embolism	240	0,2%	7 330	0,0%	1.27 (1.11 - 1.44)
**Cancer**	14 821	11,3%	396 079	1,4%	
Female breast cancer (active)	2 569	5,8%	39 062	0,1%	10.85 (10.43 - 11.30)
Female breast cancer (under surveillance)	1 066	2,4%	66 333	0,2%	2.64 (2.48 - 2.80)
Colorectal cancer (active)	1 197	0,9%	10 778	0,0%	7.00 (6.61 - 7.42)
Colorectal cancer (under surveillance)	327	0,2%	15 835	0,1%	1.38 (1.24 - 1.54)
Lung cancer (active)	1 136	0,9%	3 636	0,0%	7.83 (7.38 - 8.32)
Lung cancer (under surveillance)	259	0,2%	3 447	0,0%	2.76 (2.44 - 3.11)
Prostate cancer (active)	267	0,3%	5 343	0,0%	2.40 (2.13 - 2.70)
Prostate cancer (under surveillance)	101	0,1%	3 711	0,0%	1.31 (1.08 - 1.60)
Other cancers (active)	6 161	4,7%	103 722	0,4%	5.75 (5.60 - 5.91)

© 2025 Semenzato L et al. *JAMA Network Open*.

Other cancers (under surveillance)	2 757	2,1%	152 914	0,5%	2.16 (2.08 - 2.25)
**Inflammatory and skin diseases**					
Chronic inflammatory bowel diseases	837	0,6%	134 582	0,5%	1.17 (1.10 - 1.26)
Rheumatoid arthritis and related diseases	575	0,4%	56 479	0,2%	1.35 (1.25 - 1.47)
Ankylosing spondylitis and related diseases	693	0,5%	93 616	0,3%	0.94 (0.88 - 1.02)
**Psychological and neurodegenerative diseases**					
Neurodegenerative diseases	8 244	6,3%	268 325	0,9%	
Psychiatric disorders starting in childhood	534	0,4%	49 043	0,2%	1.81 (1.66 - 1.98)
Down syndrome	398	0,3%	7 693	0,0%	9.89 (8.96 - 10.92)
Epilepsy	3 959	3,0%	121 210	0,4%	2.22 (2.14 - 2.29)
Multiple sclerosis	835	0,6%	51 602	0,2%	2.26 (2.11 - 2.43)
Paraplegia	1 488	1,1%	28 183	0,1%	3.89 (3.69 - 4.10)
Myopathy or myasthenia gravis	562	0,4%	14 843	0,1%	3.74 (3.43 - 4.06)
Parkinson disease	314	0,2%	9 493	0,0%	2.27 (2.03 - 2.54)
Dementias (including Alzheimer's disease)	533	0,4%	4 192	0,0%	2.73 (2.51 - 2.98)
Mental impairment	1 197	0,9%	48 090	0,2%	2.15 (2.03 - 2.28)
Psycholeptic drugs (with or without a disease)	38 328	29,2%	2 150 566	7,5%	
Antidepressants	20 841	15,9%	1 387 010	4,9%	1.02 (1.00 - 1.04)
Antipsychotics	13 402	10,2%	432 754	1,5%	1.99 (1.95 - 2.03)
Anxiolytics	27 593	21,0%	1 203 644	4,2%	1.65 (1.62 - 1.68)
Hypnotics	11 652	8,9%	370 868	1,3%	1.34 (1.31 - 1.37)
**Other pathologies**					
Hemophilia or severe hemostasis disorders	214	0,2%	20 068	0,1%	1.34 (1.17 - 1.54)
HIV infection	1 385	1,1%	74 135	0,3%	1.84 (1.74 - 1.94)
Liver diseases	7 098	5,4%	111 657	0,4%	2.13 (2.08 - 2.19)
Pancreas diseases	2 359	1,8%	52 818	0,2%	1.49 (1.43 - 1.56)
Chronic dialysis	417	0,3%	2 561	0,0%	5.23 (4.74 - 5.77)
Renal transplant	383	0,3%	7 288	0,0%	3.22 (2.91 - 3.56)
**History of hospitalisation for covid-19 before November 1, 2021**	2 234	1,7%	118 748	0,4%	1.53 (1.46 - 1.59)
**Covid-19 vaccination period**					
Until July 12, 2021	75 526	57,6%	17 624 507	61,7%	1
After July 12, 2021	55 565	42,4%	10 944 391	38,3%	1.31 (1.29 - 1.32)

*This model was also adjusted for the patient's region of residence as well as their social security scheme, although these results are not presented.

eTable 3. Characteristics at Baseline of COVID-19 Vaccinated Individuals Included in the 4-Year Mortality Study by Type of First Injection Received

	mRNA-1273 vaccine		BNT162b2 vaccine	
**Characteristics**	**3 069 795**	**%**	**19 697 751**	**%**
**Age (year) - mean (std)**	39.6 (11.5)		37.7 (11.8)	
**Age**				
18 - 29	723 361	23,6%	5 830 546	29,6%
30 - 39	732 592	23,9%	4 802 416	24,4%
40 - 49	855 824	27,9%	5 033 242	25,6%
50 - 59	758 018	24,7%	4 031 547	20,5%
**Sex**				
Male	1 528 112	49,8%	9 550 831	48,5%
Female	1 541 683	50,2%	10 146 920	51,5%
**Regions**				
Ile de France	473 935	15,4%	4 081 298	20,7%
Grand Est	261 569	8,5%	1 488 950	7,6%
Hauts-de-France	305 057	9,9%	1 800 162	9,1%
Auvergne-Rhône-Alpes	323 776	10,5%	2 502 251	12,7%
Bourgogne-Franche-Comté	131 193	4,3%	768 825	3,9%
Centre-Val-de-Loire	120 859	3,9%	748 569	3,8%
Provence-Alpes-Côte d'Azur	250 512	8,2%	1 283 681	6,5%
Occitanie	305 627	10,0%	1 631 981	8,3%
Nouvelle-Aquitaine	320 979	10,5%	1 715 520	8,7%
Normandie	180 973	5,9%	971 571	4,9%
Pays de la Loire	193 175	6,3%	1 211 373	6,1%
Bretagne	181 974	5,9%	1 015 550	5,2%
Corse	6 974	0,2%	62 813	0,3%
Guadeloupe	9 658	0,3%	43 007	0,2%
Martinique	749	0,0%	46 286	0,2%
Guyane	465	0,0%	25 131	0,1%
La Réunion	1 975	0,1%	273 038	1,4%
Mayotte	345	0,0%	27 745	0,1%
**Complementary state health insurance (CSS)**	358 664	11,7%	1 728 464	8,8%
**Social deprivation index (quintiles)**				
1 (the least deprivation)	596 990	19,4%	4 276 111	21,7%
2	630 064	20,5%	4 022 173	20,4%
3	611 663	19,9%	3 739 130	19,0%
4	596 667	19,4%	3 561 607	18,1%
5 (the most deprivation)	590 759	19,2%	3 758 536	19,1%
Unknown	43 652	1,4%	340 194	1,7%
**Social security scheme**				
General social security scheme	2 778 204	90,5%	17 682 858	89,8%
MSA	126 198	4,1%	677 707	3,4%
SLM	117 723	3,8%	966 475	4,9%
Other	47 670	1,6%	370 711	1,9%
**Number of injections received on time 0**				
1	243 304	7,9%	1 603 387	8,1%
2	2 244 153	73,1%	15 234 845	77,3%
3	582 168	19,0%	2 858 469	14,5%
4	170	0,0%	1 036	0,0%
5		0,0%	12	0,0%
6		0,0%	2	0,0%
**Lifestyle habit**				
Alcohol addiction	69 964	2,3%	238 369	1,2%
Tobacco use	202 238	6,6%	940 103	4,8%
**Comorbidities**				

© 2025 Semenzato L et al. *JAMA Network Open*.

**Cardiometabolics**	347 712	11,3%	1 778 538	9,0%
Obesity	28 841	0,9%	166 021	0,8%
Diabetes	74 660	2,4%	372 384	1,9%
Lipid-lowering treatments	94 874	3,1%	446 109	2,3%
Hereditary metabolic diseases or amyloidosis	3 962	0,1%	22 638	0,1%
Hypertension	223 770	7,3%	1 118 306	5,7%
Coronary diseases	23 772	0,8%	104 590	0,5%
Obliterating arterial disease of the lower limb	8 059	0,3%	27 642	0,1%
Cardiac rhythm or conduction disturbances	13 592	0,4%	70 561	0,4%
Heart failure	4 675	0,2%	19 035	0,1%
Valvular diseases	3 672	0,1%	17 636	0,1%
Stroke	15 135	0,5%	66 108	0,3%
**Respiratory diseases**				
Chronic respiratory diseases (excluding cystic fibrosis)	121 321	4,0%	631 618	3,2%
Cystic fibrosis	213	0,0%	1 582	0,0%
Pulmonary embolism	978	0,0%	4 912	0,0%
**Cancer**	50 668	1,7%	279 814	1,4%
Female breast cancer (active)	5 066	0,2%	28 238	0,1%
Female breast cancer (under surveillance)	8 174	0,3%	47 204	0,2%
Colorectal cancer (active)	1 497	0,0%	8 007	0,0%
Colorectal cancer (under surveillance)	2 032	0,1%	11 096	0,1%
Lung cancer (active)	743	0,0%	2 816	0,0%
Lung cancer (under surveillance)	550	0,0%	2 307	0,0%
Prostate cancer (active)	771	0,0%	3 692	0,0%
Prostate cancer (under surveillance)	458	0,0%	2 581	0,0%
Other cancers (active)	13 415	0,4%	73 612	0,4%
Other cancers (under surveillance)	19 220	0,6%	106 580	0,5%
**Inflammatory and skin diseases**				
Chronic inflammatory bowel diseases	15 003	0,5%	94 288	0,5%
Rheumatoid arthritis and related diseases	6 723	0,2%	38 163	0,2%
Ankylosing spondylitis and related diseases	11 585	0,4%	63 665	0,3%
**Psychological and neurodegenerative diseases**				
Neurodegenerative diseases	38 869	1,3%	174 615	0,9%
Psychiatric disorders starting in childhood	6 777	0,2%	32 443	0,2%
Down syndrome	1 151	0,0%	4 747	0,0%
Epilepsy	18 982	0,6%	78 664	0,4%
Multiple sclerosis	5 924	0,2%	33 653	0,2%
Paraplegia	4 248	0,1%	17 456	0,1%
Myopathy or myasthenia gravis	1 922	0,1%	9 767	0,0%
Parkinson disease	1 426	0,0%	6 605	0,0%
Dementias (including Alzheimer's disease)	714	0,0%	2 755	0,0%
Mental impairment	8 520	0,3%	31 402	0,2%
Psycholeptic drugs (with or without a disease)	330 969	10,8%	1 462 452	7,4%
Antidepressants	207 891	6,8%	973 502	4,9%
Antipsychotics	80 542	2,6%	276 900	1,4%
Anxiolytics	196 658	6,4%	797 508	4,0%
Hypnotics	68 115	2,2%	242 404	1,2%
**Other pathologies**				
Hemophilia or severe hemostasis disorders	2 377	0,1%	13 444	0,1%
HIV infection	10 322	0,3%	50 031	0,3%
Liver diseases	17 284	0,6%	73 729	0,4%
Chronic hepatitis C	965	0,0%	3 561	0,0%
Pancreas diseases	7 948	0,3%	34 856	0,2%
Chronic dialysis	183	0,0%	1 692	0,0%
Renal transplant	768	0,0%	4 625	0,0%
**History of hospitalisation for covid-19**	13 317	0,4%	68 952	0,4%

© 2025 Semenzato L et al. *JAMA Network Open*.

eTable 4. Characteristics at Baseline of Individuals Included in the 4-Year Mortality Study, Dividing the Inclusion Period Into 2 Periods, ie, Before and After the Announcement of the Implementation of the Vaccine Pass on July 12, 2021

	Included until July 12, 2021				Included after July 12, 2021			
	Unvaccinated		Vaccinated		Unvaccinated		Vaccinated	
**Characteristics**	**3 791 294**	**%**	**13 908 739**	**%**	**2 141 149**	**%**	**8 858 807**	**%**
**Age (year) - mean (std)**	37.1 (11.4)		39.3 (11.7)		37.2 (11.4)		35.9 (11.6)	
**Age**								
18 - 29	1 142 397	30,1%	3 490 202	25,1%	635 385	29,7%	3 063 705	34,6%
30 - 39	1 108 945	29,2%	3 153 745	22,7%	628 124	29,3%	2 381 263	26,9%
40 - 49	843 248	22,2%	3 889 899	28,0%	479 022	22,4%	1 999 167	22,6%
50 - 59	696 704	18,4%	3 374 893	24,3%	398 618	18,6%	1 414 672	16,0%
**Sex**								
Male	1 949 903	51,4%	6 746 910	48,5%	1 106 501	51,7%	4 332 033	48,9%
Female	1 841 391	48,6%	7 161 829	51,5%	1 034 648	48,3%	4 526 774	51,1%
**Regions**								
Ile de France	677 585	17,9%	2 984 933	21,5%	387 479	18,1%	1 570 300	17,7%
Grand Est	279 496	7,4%	1 073 019	7,7%	155 777	7,3%	677 500	7,6%
Hauts-de-France	268 247	7,1%	1 283 043	9,2%	149 375	7,0%	822 176	9,3%
Auvergne-Rhône-Alpes	460 977	12,2%	1 715 148	12,3%	261 149	12,2%	1 110 879	12,5%
Bourgogne-Franche-Comté	144 911	3,8%	535 059	3,8%	81 858	3,8%	364 959	4,1%
Centre-Val-de-Loire	116 548	3,1%	543 446	3,9%	65 612	3,1%	325 982	3,7%
Provence-Alpes-Côte d'Azur	428 478	11,3%	812 482	5,8%	240 747	11,2%	721 711	8,1%
Occitanie	393 267	10,4%	1 144 121	8,2%	220 667	10,3%	793 487	9,0%
Nouvelle-Aquitaine	300 528	7,9%	1 257 166	9,0%	168 499	7,9%	779 333	8,8%
Normandie	128 009	3,4%	718 707	5,2%	70 824	3,3%	433 837	4,9%
Pays de la Loire	158 232	4,2%	878 277	6,3%	88 053	4,1%	526 271	5,9%
Bretagne	136 161	3,6%	760 582	5,5%	74 592	3,5%	436 942	4,9%
Corse	21 522	0,6%	32 465	0,2%	11 771	0,5%	37 322	0,4%
Guadeloupe	72 090	1,9%	17 968	0,1%	44 591	2,1%	34 697	0,4%
Martinique	64 950	1,7%	11 958	0,1%	39 651	1,9%	35 077	0,4%
Guyane	31 688	0,8%	11 704	0,1%	19 017	0,9%	13 892	0,2%
La Réunion	100 651	2,7%	117 002	0,8%	57 050	2,7%	158 011	1,8%
Mayotte	7 954	0,2%	11 659	0,1%	4 437	0,2%	16 431	0,2%
**Complementary state health insurance (CSS)**	788 174	20,8%	900 198	6,5%	452 389	21,1%	1 186 930	13,4%
**Social deprivation index (quintiles)**								
1 (the least deprivation)	594 913	15,7%	3 368 093	24,2%	334 511	15,6%	1 505 008	17,0%

© 2025 Semenzato L et al. *JAMA Network Open*.

2	680 133	17,9%	2 920 840	21,0%	381 631	17,8%	1 731 397	19,5%
3	719 717	19,0%	2 603 275	18,7%	405 114	18,9%	1 747 518	19,7%
4	698 532	18,4%	2 427 298	17,5%	391 229	18,3%	1 730 976	19,5%
5 (the most deprivation)	1 017 968	26,9%	2 349 849	16,9%	582 072	27,2%	1 999 446	22,6%
Unknown	80 031	2,1%	239 384	1,7%	46 592	2,2%	144 462	1,6%
**Social security scheme**		0,0%		0,0%		0,0%		0,0%
General social security scheme	3 461 410	91,3%	12 331 421	88,7%	1 953 574	91,2%	8 129 641	91,8%
MSA	133 384	3,5%	497 176	3,6%	74 137	3,5%	306 729	3,5%
SLM	108 080	2,9%	812 458	5,8%	61 877	2,9%	271 740	3,1%
Other	88 420	2,3%	267 684	1,9%	51 561	2,4%	150 697	1,7%
**Number of injections received on time 0**								
1		0,0%	1 071 658	7,7%		0%	775 033	8,7%
2		0,0%	12 336 476	88,7%		0%	5 142 522	58,0%
3		0,0%	500 196	3,6%		0%	2 940 441	33,2%
4		0,0%	406	0,0%		0%	800	0,0%
5		0,0%	2	0,0%		0%	10	0,0%
6		0,0%	1	0,0%		0%	1	0,0%
**Lifestyle habit**								
Alcohol addiction	58 272	1,5%	159 412	1,1%	31 456	1,5%	148 921	1,7%
Tobacco use	173 855	4,6%	687 148	4,9%	95 977	4,5%	455 193	5,1%
**Comorbidities**								
**Cardiometabolics**	297 440	7,8%	1 427 469	10,3%	167 156	7,8%	698 781	7,9%
Obesity	25 366	0,7%	123 242	0,9%	14 130	0,7%	71 620	0,8%
Diabetes	75 784	2,0%	288 483	2,1%	42 781	2,0%	158 561	1,8%
Lipid-lowering treatments	66 207	1,7%	381 947	2,7%	37 167	1,7%	159 036	1,8%
Hereditary metabolic diseases or amyloidosis	3 726	0,1%	17 417	0,1%	2 155	0,1%	9 183	0,1%
Hypertension	180 772	4,8%	913 730	6,6%	101 512	4,7%	428 346	4,8%
Coronary diseases	23 672	0,6%	84 439	0,6%	13 203	0,6%	43 923	0,5%
Obliterating arterial disease of the lower limb	7 124	0,2%	21 733	0,2%	3 936	0,2%	13 968	0,2%
Cardiac rhythm or conduction disturbances	14 462	0,4%	54 009	0,4%	7 966	0,4%	30 144	0,3%
Heart failure	5 376	0,1%	14 417	0,1%	2 989	0,1%	9 293	0,1%
Valvular diseases	4 069	0,1%	13 757	0,1%	2 262	0,1%	7 551	0,1%
Stroke	15 562	0,4%	51 171	0,4%	8 840	0,4%	30 072	0,3%
Pulmonary embolism	1 099	0,0%	3 610	0,0%	581	0,0%	2 280	0,0%
**Respiratory diseases**								
Chronic respiratory diseases (excluding cystic fibrosis)	117 536	3,1%	476 355	3,4%	65 601	3,1%	276 584	3,1%

© 2025 Semenzato L et al. *JAMA Network Open*.

Cystic fibrosis	314	0,0%	1 170	0,0%	177	0,0%	625	0,0%
**Cancer**	51 633	1,4%	220 019	1,6%	28 785	1,3%	110 463	1,2%
Female breast cancer (active)	5 370	0,1%	22 362	0,2%	2 957	0,1%	10 942	0,1%
Female breast cancer (under surveillance)	7 695	0,2%	38 950	0,3%	4 326	0,2%	16 428	0,2%
Colorectal cancer (active)	1 591	0,0%	6 362	0,0%	880	0,0%	3 142	0,0%
Colorectal cancer (under surveillance)	1 905	0,1%	9 005	0,1%	1 129	0,1%	4 123	0,0%
Lung cancer (active)	783	0,0%	2 170	0,0%	430	0,0%	1 389	0,0%
Lung cancer (under surveillance)	520	0,0%	1 860	0,0%	329	0,0%	997	0,0%
Prostate cancer (active)	733	0,0%	3 120	0,0%	414	0,0%	1 343	0,0%
Prostate cancer (under surveillance)	511	0,0%	2 158	0,0%	262	0,0%	881	0,0%
Other cancers (active)	14 701	0,4%	56 211	0,4%	8 155	0,4%	30 816	0,3%
Other cancers (under surveillance)	19 185	0,5%	82 845	0,6%	10 686	0,5%	42 955	0,5%
**Inflammatory and skin diseases**								
Chronic inflammatory bowel diseases	16 780	0,4%	70 971	0,5%	9 348	0,4%	38 320	0,4%
Rheumatoid arthritis and related diseases	7 741	0,2%	28 931	0,2%	4 427	0,2%	15 955	0,2%
Ankylosing spondylitis and related diseases	12 179	0,3%	49 011	0,4%	6 880	0,3%	26 239	0,3%
**Psychological and neurodegenerative diseases**								
Neurodegenerative diseases	40 586	1,1%	127 336	0,9%	22 499	1,1%	86 148	1,0%
Psychiatric disorders starting in childhood	6 625	0,2%	20 607	0,1%	3 732	0,2%	18 613	0,2%
Down syndrome	1 434	0,0%	3 738	0,0%	759	0,0%	2 160	0,0%
Epilepsy	17 816	0,5%	55 221	0,4%	9 707	0,5%	42 425	0,5%
Multiple sclerosis	8 243	0,2%	24 638	0,2%	4 617	0,2%	14 939	0,2%
Paraplegia	5 093	0,1%	13 191	0,1%	2 874	0,1%	8 513	0,1%
Myopathy or myasthenia gravis	2 374	0,1%	7 386	0,1%	1 342	0,1%	4 303	0,0%
Parkinson disease	1 143	0,0%	5 481	0,0%	633	0,0%	2 550	0,0%
Dementias (including Alzheimer's disease)	837	0,0%	2 007	0,0%	419	0,0%	1 462	0,0%
Mental impairment	6 051	0,2%	25 183	0,2%	3 314	0,2%	14 739	0,2%
Psycholeptic drugs (with or without a disease)	254 193	6,7%	1 144 358	8,2%	141 280	6,6%	649 063	7,3%
Antidepressants	145 630	3,8%	777 317	5,6%	80 828	3,8%	404 076	4,6%
Antipsychotics	57 237	1,5%	207 789	1,5%	31 477	1,5%	149 653	1,7%
Anxiolytics	152 442	4,0%	615 124	4,4%	84 629	4,0%	379 042	4,3%
Hypnotics	46 352	1,2%	189 542	1,4%	25 649	1,2%	120 977	1,4%
**Other pathologies**								
Hemophilia or severe hemostasis disorders	2 805	0,1%	9 646	0,1%	1 656	0,1%	6 175	0,1%
HIV infection	9 685	0,3%	36 190	0,3%	5 482	0,3%	24 163	0,3%
Liver diseases	17 776	0,5%	52 088	0,4%	9 966	0,5%	38 925	0,4%
Chronic hepatitis C	1 107	0,0%	2 554	0,0%	609	0,0%	1 972	0,0%

© 2025 Semenzato L et al. *JAMA Network Open*.

Pancreas diseases	7 949	0,2%	24 756	0,2%	4 424	0,2%	18 048	0,2%
Chronic dialysis	711	0,0%	876	0,0%	392	0,0%	999	0,0%
Renal transplant	1 450	0,0%	3 213	0,0%	828	0,0%	2 180	0,0%
**History of hospitalisation for covid-19**	22 670	0,6%	34 957	0,3%	16 043	0,7%	47 312	0,5%

© 2025 Semenzato L et al. *JAMA Network Open*.

eTable 5. Number of Deceased Patients by Vaccination Status in the Study of Short-Term Mortality

	Cause of death				
**Vaccination schedule**	**All-cause**	**Tumor**	**Circulatory diseases**	**External causes**	**COVID-19**
**Unvaccinated**	21 693	6 089	2 036	3 612	1 382
**After dose 1**					
Risk period - In the 3 months	2 894	742	340	622	93
Risk period - Between 3 and 6 months	1047	390	101	161	17
Control period – after 6 months	635	235	65	91	10
**After dose 2**					
Risk period - In the 3 months	6 634	1 907	749	1 357	43
Risk period - Between 3 and 6 months	7 957	2 879	844	1 285	98
Control period - after 6 months	5 329	2 231	436	727	104
**After dose 3**					
Risk period - In the 3 months	5 398	1 594	657	1 082	72
Risk period - Between 3 and 6 months	6 308	2 204	638	1 121	80
Control period - after 6 months	2 699	1 154	245	335	69
**After dose 4**					
Risk period - In the 3 months	198	75	16	8	18
Risk period - Between 3 and 6 months	140	74	13	3	9
Control period - after 6 months	47	22	5	2	4

The few events occurring on the day of vaccination were considered separately.

© 2025 Semenzato L et al. *JAMA Network Open*.

eTable 6. Relative Incidence of Short-Term Mortality, All Causes, by Cancer, External Causes, Circulatory Diseases, and COVID-19, Within 2 subperiods of 3 Months Following Vaccination, Using Adapted SCCS Models

	**Cause of death**				
**Risk window**	**All-cause**	**Tumor**	**Circulatory diseases**	**External causes**	**COVID-19**
After dose 1					
Risk period - In the 3 months	0.60 [0.58-0.62]	0.56 [0.52-0.61]	0.66 [0.58-0.74]	0.66 [0.60-0.73]	0.66 [0.50-0.88]
Risk period - Between 3 and 6 months	0.50 [0.47-0.54]	0.68 [0.61-0.76]	0.46 [0.37-0.57]	0.41 [0.35-0.49]	0.14 [0.08-0.25]
After dose 2					
Risk period - In the 3 months	0.60 [0.57-0.62]	0.58 [0.54-0.62]	0.66 [0.58-0.75]	0.70 [0.63-0.78]	0.13 [0.09-0.18]
Risk period - Between 3 and 6 months	0.74 [0.71-0.77]	0.86 [0.81-0.91]	0.74 [0.65-0.84]	0.76 [0.68-0.84]	0.29 [0.22-0.38]
After dose 3					
Risk period - In the 3 months	0.61 [0.58-0.64]	0.58 [0.54-0.63]	0.70 [0.59-0.83]	0.83 [0.71-0.97]	0.17 [0.12-0.24]
Risk period - Between 3 and 6 months	0.85 [0.81-0.89]	0.89 [0.83-0.96]	0.76 [0.65-0.90]	0.97 [0.84-1.11]	0.50 [0.37-0.69]
After any dose					
Risk period - In the 3 months	0.58 [0.56-0.59]	0.56 [0.53-0.59]	0.64 [0.57-0.71]	0.64 [0.58-0.69]	0.29 [0.23-0.37]
Risk period - Between 3 and 6 months	0.71 [0.69-0.73]	0.83 [0.79-0.87]	0.68 [0.61-0.75]	0.66 [0.61-0.72]	0.40 [0.32-0.50]

The few events occurring on the day of vaccination were considered separately.

© 2025 Semenzato L et al. *JAMA Network Open*.
