# Supplementary material for: COVID-19 mRNA Vaccination and 4-Year All-Cause Mortality Among Adults Aged 18 to 59 Years in France
Source: JAMA Netw Open. 2025 Dec 4;8(12):e2546822. doi: 10.1001/jamanetworkopen.2025.46822 (PMC12679329; doi:10.1001/jamanetworkopen.2025.46822)
Supplement: Supplement 2. — Data Sharing Statement [file jamanetwopen-e2546822-s002.pdf]

Data Sharing Statement

Semenzato. COVID-19 mRNA Vaccination and 4-Year All-Cause Mortality Among Adults Aged 18 to 59 Years in France. *JAMA Netw Open*. Published December 04, 2025. doi:10.1001/jamanetworkopen.2025.46822

Data
Data available: No
Additional Information:EPI-PHARE has direct access to the SNDS from the permanent regulatory access of its constitutive bodies, the French National Agency for the Safety of Medicines (ANSM) and Health Products and the French National Health Insurance (Cnam). Permanent access is given according to French Decree No. 2016-1871 of December 26, 2016, relating to the processing of personal data called the “National Health Data System” and French law articles Art. R. 1461-13 and 1461-14. All requests in the database were made by duly authorized people and this study was declared prior to its initiation on the EPI-PHARE registry of studies requiring the SNDS (number T-2023-01-446).
